# BRCA1-regulated RRM2 expression protects glioblastoma cells from endogenous replication stress and promotes tumorigenicity

**DOI:** 10.1038/ncomms13398

**Published:** 2016-11-15

**Authors:** Rikke D. Rasmussen, Madhavsai K. Gajjar, Lucie Tuckova, Kamilla E. Jensen, Apolinar Maya-Mendoza, Camilla B. Holst, Kjeld Møllgaard, Jane S. Rasmussen, Jannick Brennum, Jiri Bartek, Martin Syrucek, Eva Sedlakova, Klaus K. Andersen, Marie H. Frederiksen, Jiri Bartek, Petra Hamerlik

**Affiliations:** 1 Brain Tumor Biology, Danish Cancer Society Research Center, Strandboulevarden 49, Copenhagen DK-2100, Denmark; 2 Department of Clinical and Molecular Pathology, Faculty of Medicine and Dentistry, Palacky University and University Hospital Olomouc, Hnevotinska 3, Olomouc 77515, Czech Republic; 3 Genome Integrity Unit, Danish Cancer Society Research Center, Strandboulevarden 49, Copenhagen DK-2100, Denmark; 4 Department of Cellular and Molecular Medicine, Faculty of Health and Medical Sciences, University of Copenhagen, Blegdamsvej 3, Copenhagen 2200-DK, Denmark; 5 Department of Neurosurgery, Copenhagen University Hospital, Blegdamsvej 9, Copenhagen DK-2100, Denmark; 6 Department of Medicine, Unit of Microbial Pathogenesis, Karolinska Institutet and Department of Neurosurgery, Karolinska University Hospital, Solna 171 76, Stockholm, Sweden; 7 Department of Pathology, Hospital Na Homolce, Roentgenova 2, 150 30 Praha 5, Czech Republic; 8 Statistics, Bioinformatics and Registry Unit, Danish Cancer Society Research Center, Strandboulevarden 49, Copenhagen DK-2100, Denmark; 9 Division of Translational Medicine and Chemical Biology, Department of Medical Biochemistry and Biophysics, Science for Life Laboratory, Karolinska Institute, Scheeles vag 2, Stockholm 17177, Sweden; 10 Department of Radiation Biology, Copenhagen University Hospital, Blegdamsvej 9, Copenhagen 2100-DK, Denmark

## Abstract

Oncogene-evoked replication stress (RS) fuels genomic instability in diverse cancer types. Here we report that BRCA1, traditionally regarded a tumour suppressor, plays an unexpected tumour-promoting role in glioblastoma (GBM), safeguarding a protective response to supraphysiological RS levels. Higher BRCA1 positivity is associated with shorter survival of glioma patients and the abrogation of BRCA1 function in GBM enhances RS, DNA damage (DD) accumulation and impairs tumour growth. Mechanistically, we identify a novel role of BRCA1 as a transcriptional co-activator of *RRM2* (catalytic subunit of ribonucleotide reductase), whereby BRCA1-mediated RRM2 expression protects GBM cells from endogenous RS, DD and apoptosis. Notably, we show that treatment with a RRM2 inhibitor triapine reproduces the BRCA1-depletion GBM-repressive phenotypes and sensitizes GBM cells to PARP inhibition. We propose that GBM cells are addicted to the RS-protective role of the BRCA1-RRM2 axis, targeting of which may represent a novel paradigm for therapeutic intervention in GBM.

## Abstract (web summary)

BRCA1 loss can result in collapse of replication forks into DNA double strand breaks that can contribute to malignant transformation. Here, the authors find that BRCA1 promotes the expression of RRM2 protecting glioblastoma cells from replication stress, DNA damage and apoptosis.

Faithful completion of chromosomal DNA replication is essential for genome integrity. Replication stress (RS) including stalling or collapse of replication forks can be induced by activated oncogenes and numerous cancer chemotherapeutics. Exposure to genotoxic insults results in activation of checkpoint cascades that impose cell-cycle arrest thereby preventing propagation of damaged DNA. During S phase, the genome is replicated through a fundamental process that requires spatio-temporal coordination of many replication origins. The intra-S phase checkpoint responds to replication-associated DNA damage and suppresses firing of new origins, inhibits elongation and stabilizes ongoing replication forks to avoid genome destabilization and carcinogenesis^1^. BRCA1 is a tumour suppressor implicated in DNA repair, transcription, chromatin remodelling and cell survival. In mammalian cells, Fanconi anaemia and tumour suppressor BRCA1/2 proteins protect the replication forks. These proteins stabilize nucleoprotein filaments composed of RAD51 and nascent single stranded DNA (ssDNA) at stalled forks, thereby preventing MRE11 nuclease-mediated DNA strand degradation^2^,^3^. Human replication protein A (RPA) is a highly conserved ssDNA-binding protein that plays critical roles in DNA replication and repair^4^. RPA accumulates on ssDNA at stalled and collapsed forks, thereby providing a signal for activation of the intra-S checkpoint^5^. In S phase, RPA co-localizes with Rad51, a protein thought to remove RPA during formation of a nucleoprotein complex during homologous recombination DNA repair (HR)^6^. RPA phosphorylation, increased foci formation by RPA/Rad51 in S-phase cells, and the induction of 53BP1 ‘bodies’ in the following G1 phase represent hallmarks of ongoing RS (refs 7, 8, 9). BRCA1 loss can result in collapse of replication forks into DNA double strand breaks (DSBs)^2^,^10^,^11^ that can contribute to malignant transformation. DSBs trigger the DNA damage response (DDR) network including checkpoints that provide an intrinsic barrier to carcinogenesis^12^,^13^. BRCA1 is expressed in many adult mostly proliferative tissues^14^, and its loss can induce apoptosis^15^,^16^,^17^,^18^. *BRCA1* gene resides on human chromosome 17q21 (ref. 16), and germ-line *BRCA1* mutations account for large subsets of hereditary breast and ovarian cancer cases^16^,^17^. Reflecting the concept of synthetic lethality BRCA1 and BRCA2-defective tumours are intrinsically sensitive to Poly (ADP-ribose) polymerase (PARP) inhibitors^18^,^19^. PARP inhibitors (PARPi) cause accumulation of single-strand DNA breaks (SSBs), which are then converted into irreparable cytotoxic DSBs in BRCA1/2-defective cells^20^. Interestingly, even some tumours with intact *BRCA1/2* may exhibit sensitivity to PARPi, such as glioblastomas (GBM), where treatment with olaparib (a PARP inhibitor) showed promising results in pre-clinical^21^,^22^ and phase I clinical studies (https://clinicaltrials.gov). Prognosis of GBM (WHO grade IV glioma)^23^ patients; however, remains dismal with median survival of only ∼15 months^24^. Several studies including ours showed that malignant gliomas exhibit constitutive activation of the DDR, a network whose various facets have been implicated in early-stage protection against tumour progression^25^,^26^, yet also tumour maintenance and therapeutic resistance in later-stage cancers^23^. Given the pronounced genomic instability and endogenous RS in gliomas, we reasoned that these tumours may develop dependence on BRCA1, a hypothesis tested in the present study. Indeed, here we show that BRCA1 is a negative prognostic factor for glioma patient survival. Furthermore, we identify BRCA1 as a transcriptional regulator of *RRM2*, a gene encoding the catalytic subunit of ribonucleotide reductase (RNR), which protects GBM cells from endogenous RS and thereby promotes their survival and tumorigenic potential. Importantly, triapine-mediated inhibition of RRM2 or BRCA1 depletion impaired growth of GBM cells and, moreover, sensitized GBMs to olaparib. Therefore, triapine alone or in combination with olaparib may represent a novel therapeutic intervention in malignant gliomas.

## Results

### BRCA1 is essential for GBM cell viability and tumour growth

**Figure 1:**
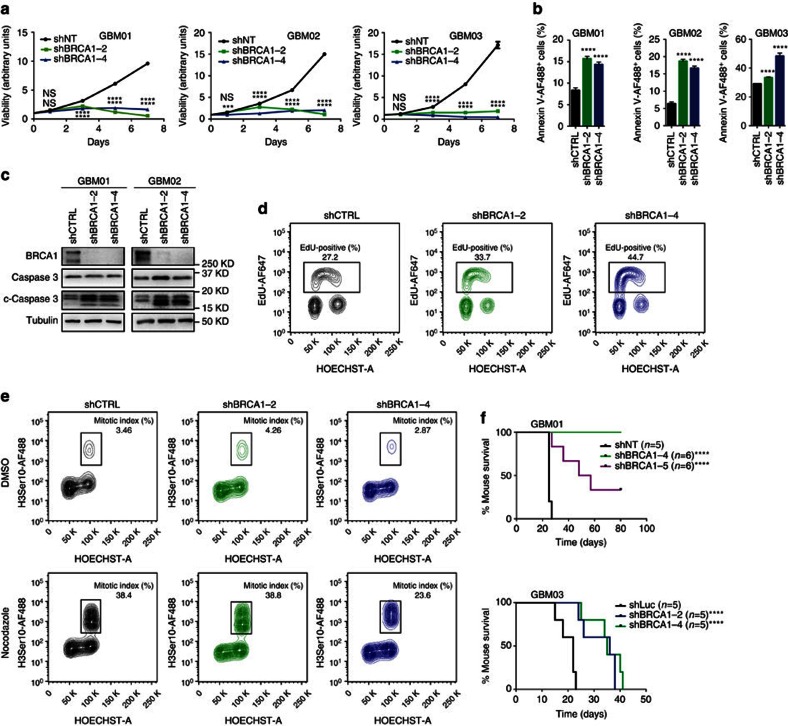
BRCA1 is essential for GBM cell maintenance. (**a**) Viability curves for GBM01, GBM02 and GBM03 cells transduced with shCTRL virus or two independent BRCA1-targeting shRNAs (shBRCA1-2 and shBRCA1-4) (7 days). (**b**) Quantification of apoptotic cells (% of Annexin V-positive) for GBM01, GBM02 and GBM03 cells transduced with shCTRL virus or two independent BRCA1-targeting shRNAs (shBRCA1-2 and shBRCA1-4). (**c**) Immunoblot analysis of BRCA1, caspase 3 and cleaved caspase-3 in GBM01 and GBM02 cells transduced with shCTRL virus or two independent BRCA1-targeting shRNAs (shBRCA1-2 and shBRCA1-4). Tubulin was used as a loading control. (**d**) FACS analysis of cell cycle profile and the proliferative index (% of Edu-positive cells) in GBM01 cells transduced with shCTRL virus or two independent BRCA1-targeting shRNAs (shBRCA1-2 and shBRCA1-4). (**e**) FACS analysis of cell cycle profile and the mitotic index (% of H3^Ser10^-positive cells) in DMSO or nocodazole treated GBM01 cells transduced with shCTRL virus or two independent BRCA1-targeting shRNAs (shBRCA1-2 and shBRCA1-4). (**f**) Kaplan-Meier survival curve for NMRI nude mice intracranially injected with GBM01 or GBM03 cells transduced with shCTRL virus or two independent BRCA1-targeting shRNAs (shBRCA1-2 and shBRCA1-4 or shBRCA1-5). Statistical significance was calculated by one-way ANOVA, Tukey’s multiple comparisons test (*in vitro* study) and Log-rank/Mantel-Cox test (*in vivo* study). All data are shown as means±s.d. and performed as technical triplicates. (*P<0.05, ^**^P<0.005, ^***^P<0.005, ^****^P<0.0001; NS represents non-significance).

To explore the biological significance of BRCA1 in GBM cells (for baseline BRCA1 expression levels in cell models used here, see Supplementary Fig. 1e), we performed shRNA-mediated knockdown of BRCA1 in three representative GBM xenograft lines and measured their viability over a period of seven days. BRCA1 knockdown impaired GBM cell viability, increased apoptosis (Fig. 1a–c) and caused a notable accumulation in S phase (Fig. 1d). To assess the cell-cycle checkpoint integrity we used the mitotic inhibitor nocodazole to trap cells at their exit from mitosis. GBM cells with BRCA1 knockdown were partially arrested at G2 phase and exhibited only modest reduction (compared with DMSO-treated controls) of G2/M checkpoint delay after nocodazole treatment (Fig. 1e). BRCA1 knockdown in normal human astrocytes (NHA-26 and NHA-DRB) also decreased their viability and caused G1 arrest (Supplementary Fig. 1a,c). To investigate whether the effect of BRCA1 knockdown on cell viability is unique to GBM cancer cells, we have tested additional four cancer cell lines. BRCA1 loss in Cal51 (breast carcinoma) and OVCAR5 (ovarian carcinoma) cells did neither impair their viability, nor affected cell cycle progression (Supplementary Fig. 2a,b). Prostate cancer cell line PC3 and cervical carcinoma cell line HELA encountered decreased viability, but continued growing over a period of seven days (Supplementary Fig. 2a). The cell cycle analysis of PC3 cells lacking BRCA1 showed significant reduction on S-phase accompanied by G2/M arrest (Supplementary Fig. 2b). In non-malignant control cells (BJ; human foreskin fibroblasts), BRCA1 knockdown reduced their viability and induced G2/M arrest (Supplementary Fig. 1b,c).

To further demonstrate the impact of BRCA1 loss in gliomagenesis, we orthotopically injected two representative GBM xenograft lines; each transduced either with control shRNA (shCTRL) or two non-overlapping shRNAs targeting BRCA1 into immunocompromised mice. BRCA1 knockdown significantly extended survival of the tumour-bearing mice (Fig. 1f), thereby confirming the supporting role of BRCA1 in GBM growth and maintenance.

### BRCA1 protects GBM cells from RS-induced DSB formation

**Figure 2:**
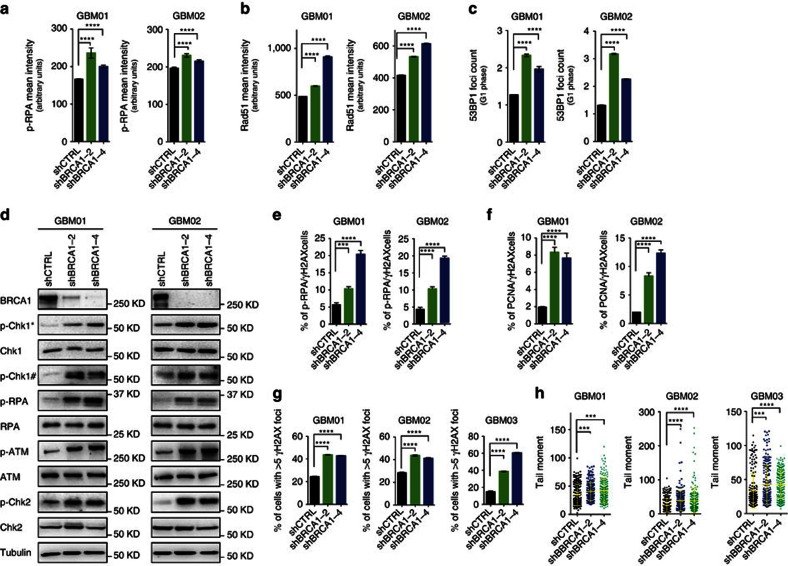
BRCA1 protects GBM cells from replication stress-induced DSBs formation. (**a**) Microscopy analysis and quantification of pRPA mean signal intensity (in S phase) in GBM cells transduced with shCTRL or two independent BRCA1-targeting shRNAs (shBRCA1-2 and shBRCA1-4). (**b**) Microscopy analysis and quantification of Rad51 mean signal intensity (S phase) in GBM cells transduced with shCTRL or two independent BRCA1-targeting shRNAs (shBRCA1-2 and shBRCA1-4). (**c**) Microscopy analysis and quantification of 53BP1 foci count (G1 phase) in GBM cells transduced with shCTRL or two independent BRCA1-targeting shRNAs (shBRCA1-2 and shBRCA1-4). (**d**) Immunoblot analysis of DDR activation in GBM cells transduced with shCTRL or two independent BRCA1-targeting shRNAs (shBRCA1-2 and shBRCA1-4). * Chk1Ser317; # Chk1Ser345. (**e**) FACS quantification of double-positive p-RPA/γH2AX GBM cells transduced with shCTRL or two independent BRCA1-targeting shRNAs (shBRCA1-2 and shBRCA1-4). (**f**) FACS quantification of double-positive PCNA/γH2AX GBM cells transduced with shCTRL or two independent BRCA1-targeting shRNAs (shBRCA1-2 and shBRCA1-4). (**g**) Microscopy analysis and quantification of γH2AXfoci count in GBM cells transduced with shCTRL or two independent BRCA1-targeting shRNAs (shBRCA1-2 and shBRCA1-4). (**h**) Comet assay and tail moment quantification of DSBs in GBM cells transduced with shCTRL or two independent BRCA1-targeting shRNAs (shBRCA1-2 and shBRCA1-4). Statistical significance was calculated by one-way ANOVA and Tukey’s multiple comparisons test in **a**–**c**,**e**–**h** and all data are shown as means ±s.d. and performed as technical triplicates. (*P<0.05, ^**^P<0.005, ^***^P<0.005, ^****^P<0.0001; NS represents non-significance).

To understand the cause of S-phase arrest after BRCA1 knockdown in GBM cells, indicative of enhanced RS levels, we employed several methods to evaluate the extent of RS-induced DNA damage and DDR activation. Microscopy analyses of GBM01 and GBM02 cells confirmed increased p-RPA/Rad51 foci in S phase and 53BP1 body counts in G1 after shRNA-mediated BRCA1 knockdown (shBRCA1-2/-4) compared with control (shCTRL) (Fig. 2a–c). DDR activation is thought to reflect DNA RS (refs 12, 13, 27, 28) and, consistently, BRCA1 knockdown in GBM cells led to the activation of ATM/Chk2-Chk1/RPA signalling (Fig. 2d). In addition, we performed flow cytometry analysis (FACS) of cells double-stained for p-RPA^+^/ γH2AX^+^or PCNA^+^/γH2AX^+^, to evaluate the frequency of S-phase cells experiencing enhanced RS. Both, the fraction of PCNA^+^/γH2AX^+^and p-RPA^+^/ γH2AX^+^cells were increased after BRCA1 knockdown compared with controls, indicative of replication fork stalling and/or collapse into DSBs (Fig. 2e,f). Microscopy-based analysis of γH2AX foci counts and comet assay confirmed significant induction of DSBs in GBM cells lacking BRCA1 (shBRCA1-2/-4) (Fig. 2g,h). Altogether, our data indicate that BRCA1 facilitates protection of GBM cells against endogenous RS-induced DNA damage. Notably, the immunoblot analysis of p-RPA in normal human controls (NHA-DRB, BJ) and the four non-GBM cancer cell lines (OVCAR5, Cal51, PC3 and HELA) confirmed elevated p-RPA levels after BRCA1 knockdown (Supplementary Figs 1d and 2c).

### BRCA1-regulated RRM2 protects GBM from replication stress

**Figure 3:**
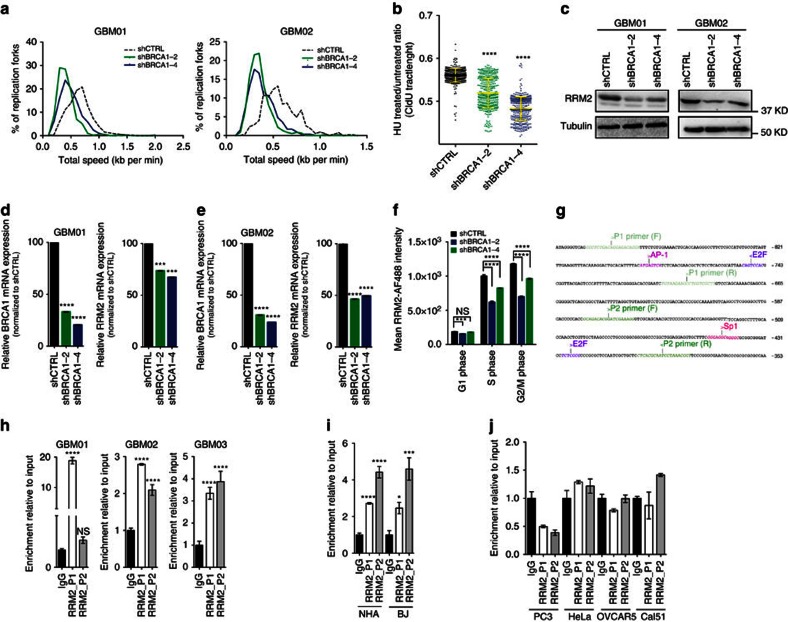
BRCA1 loss impedes replication fork progression by down-regulating RRM2 in GBM cells. (**a**) DNA fibre assay measuring the replication fork progression speed in GBM cells (GBM01 and GBM02) transduced with shCTRL or two independent BRCA1-targeting shRNAs (shBRCA1-2 and shBRCA1-4). See also Table 1. (**b**) Replication fork recovery assay showing the quantification of CldU tract length in GBM01 cells transduced with shCTRL or 2 non-overlapping BRCA1-targeting shRNAs (shBRCA1-2 and shBRCA1-4) and treated or not with 2 mM HU (4 h) prior CldU labelling. See also Table 2; Supplementary Table 1. (**c**) Immunoblot analysis of RRM2 protein levels in GBM cells transduced with shCTRL or two independent BRCA1-targeting shRNAs (shBRCA1-2 and shBRCA1-4). (**d**,**e**) RT-qPCR analysis of BRCA1 and RRM2 mRNA levels in GBM cells transduced with shCTRL or two independent BRCA1-targeting shRNAs (shBRCA1-2 and shBRCA1-4). (**f**) FACS analysis of RRM2 protein level changes throughout cell cycle in GBM cells (GBM01) transduced with shCTRL or two independent BRCA1-targeting shRNAs (shBRCA1-2 and shBRCA1-4). (**g**) Partial sequence of the human RRM2 promoter (GenBank accession number AY032750)^33^, which was used to design Chip primers: P1 primer forward (F)/reverse (R) and P2 primer forward (F)/reverse (R). Positions are numbered from the downstream transcription initiation site (+1). Putative binding sites for transcription factors are color-coded and identified above the sequence. (**h**) Chip immunoprecipitation of BRCA1 binding RRM2 promoter in GBM01-03 cells using primer set P1 and P2. (**i**) Chip immunoprecipitation of BRCA1 binding RRM2 promoter in NHA-DRB and BJ cells using primer set P1 and P2. (**j**) Chip immunoprecipitation of BRCA1 binding RRM2 promoter in PC3, HELA, OVCAR and Cal51 cells using primer set P1 and P2. Statistical significance was calculated by one-way ANOVA and Tukey’s multiple comparisons test in **d**–**f**,**h**–**j** or Student’s *t* test (**a**,**b**) and all data are shown as means±s.d. and performed as technical triplicates. (*P<0.05, ^**^P<0.005, ^***^P<0.005, ^****^P<0.0001; NS represents non-significance).

Using DNA fibre assays, BRCA1 was shown to protect replication fork stability and progression^2^,^11^. Here, BRCA1 knockdown negatively impacted the total fork speed (Fig. 3a and Table 1). To test the involvement of BRCA1 in the recovery of stalled replication forks, we monitored the stability of nascent replication tracts^29^ post exposure to exogenous RS (2 mM hydroxyurea (HU) for 4 h). Specifically, nascent replication tracts were CldU-labelled after replication stalling with HU. HU treatment resulted in markedly shorter CldU tract length in cells with BRCA1 knockdown compared with control (Fig. 3b, Table 2 and Supplementary Table 1). These data confirmed the importance of BRCA1 in regulating the replication fork speed and fork recovery after stalling in GBM cells. The HU-induced CldU tract shortening observed even in the shCTRL-exposed cells indicated that GBM cells are sensitive to dNTP depletion already in the presence of BRCA1 (Table 2 and Supplementary Table 1), and this phenotype was further enhanced on BRCA1 knockdown. HU depletes cellular deoxyribonucleotide triphosphate (dNTP) levels via inhibition of ribonucleotide reductase (RNR), an enzyme which plays a key role in regulating DNA replication, and consequently, cell proliferation^30^. RNR is responsible for dNTPs biogenesis and consists of two large catalytic subunits (RRM1) and two small regulatory subunits (RRM2). Of importance, RRM2 is the regulatory subunit controlling dNTP synthesis during S phase of the cell cycle and is rate-limiting for RNR activity^31^. Thus, we sought to determine whether RRM2 expression is regulated by BRCA1. Notably, both the messengerRNA (mRNA) and protein levels of RRM2 were decreased in GBM cells lacking BRCA1 (Fig. 3c–e), indicating that BRCA1 may function as a transcriptional regulator of RRM2 expression. To exclude the possibility that this decrease in RRM2 levels is just a consequence of cell cycle arrest invoked by BRCA1 knockdown, we have performed additional analysis of RRM2 protein levels in individual cell cycle phases using flow cytometry analysis. As shown in Fig. 3f, RRM2 levels were increasing as the GBM01-shCTRL cells progressed from G1 through S phase and peaked at G2/M phase. In GBM01 cells with BRCA1 knockdown (shBRCA1-2 & shBRCA1-4), the RRM2 protein levels significantly decreased in all cell cycle phases, while this decrease was more prominent in S-G2/M phases than in G1 phase.

To test our hypothesis that BRCA1 acts as a transcriptional co-activator of RRM2, we performed chromatin immunoprecipitation (ChIP) using BRCA1 antibody. Using two independent primer sets (P1 & P2, see Fig. 3g), we confirmed BRCA1 recruitment to *RRM2* promoter region in GBM01, GBM02, as well as GBM03 cells (Fig. 3h), thereby identifying a novel role of BRCA1 as an upstream regulator of RRM2. Using the same approach, we have confirmed BRCA1 binding to RRM2 promoter in NHA-DRB and BJ cells (Fig. 3i), but not in non-GBM cancer cell lines PC3, HELA; OVCAR5 or Cal51 (Fig. 3j). Intriguingly, BRCA1 knockdown did not result in RRM2 protein level changes in either NHA-DRB or BJ cells (Supplementary Fig. 1d).

**Figure 4:**
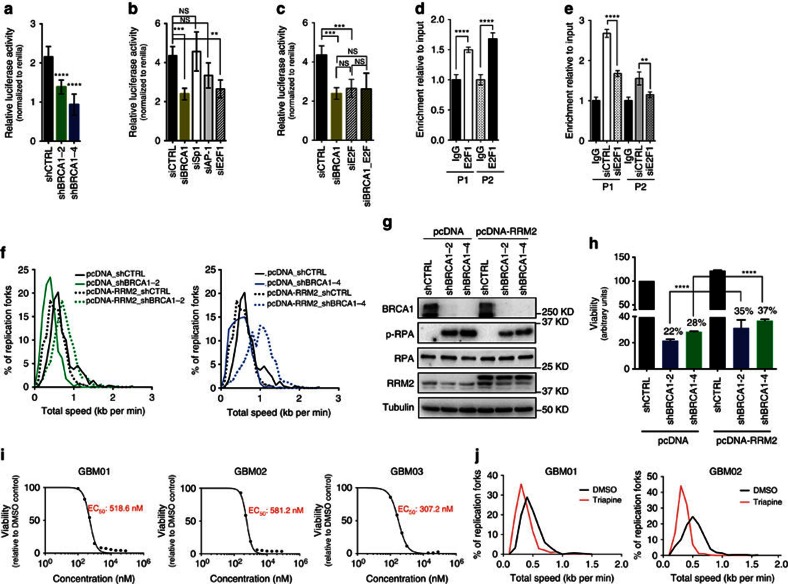
BRCA1 regulates RRM2 expression via E2F1 and RRM2 overexpression rescues BRCA1-loss phenotype in GBM cells. (**a**) Luciferase assay of transcriptional activation of the RRM2 promoter in GBM01 transduced with shCTRL or shBRCA1-2/shBRCA1-4. (**b**) Luciferase assay of transcriptional activation of the RRM2 promoter in GBM01 after siRNA-mediated knockdown of Sp1; AP-1 and E2F1 in comparison to BRCA1. (**c**) Luciferase assay of transcriptional activation of the RRM2 promoter in GBM01 after siRNA-mediated knockdown of BRCA1 and E2F1 alone or on combination. (**d**) Chip immunoprecipitation of E2F1 binding RRM2 promoter in GBM01 cells using primer set P1 and P2. (**e**) Chip immunoprecipitation of BRCA1 binding RRM2 promoter in GBM01 cells transfected with siCTRL and siE2F1 using primer set P1 and P2. (**f**) DNA fibre assay measuring the replication fork progression speed in GBM cells lacking BRCA1 (shBRCA1-2 and shBRCA1-4) or not (shCTRL) transfected with either control vector (pcDNA) or vector expressing RRM2 (pcDNA-RRM2). See also Table 3. (**g**) Cell viability of GBM cells lacking BRCA1 (shBRCA1-2 and shBRCA1-4) or not (shCTRL) transfected with either control vector (pcDNA) or vector expressing RRM2 (pcDNA-RRM2). (**h**) Immunoblot analysis of BRCA1, p-RPA, RPA and RRM2 in GBM cells lacking BRCA1 (shBRCA1-2 and shBRCA1-4) or not (shCTRL) transfected with either control vector (pcDNA) or vector expressing RRM2 (pcDNA-RRM2). (**i**) Viability-based assessment of EC_50_ triapine concentrations in GBM01, GBM02 and GBM03 cells. (**j**) DNA fibre assay measuring the replication fork progression speed in GBM cells treated with DMSO or EC_50_ triapine. See also Table 4. Statistical significance was calculated by one-way ANOVA and Tukey’s multiple comparisons test in **a**–**d**,**f**,**h** or Student’s *t* test (**e**,**i**) and all data are shown as means ±s.d. and performed as technical triplicates. (*P<0.05, ^**^P<0.005, ^***^P<0.005, ^****^P<0.0001; NS represents non-significance).

In addition to ChIP, we have employed luciferase reporter assay to measure transcriptional activation of RRM2 promoter in GBM01 cells. In comparison to shCTRL, BRCA1 knockdown (shBRCA1-2/shBRCA1-4) significantly reduced transcriptional activity of RRM2 promoter in GBM01 cells (Fig. 4a). A role for BRCA1 as transcription factor is well established, but given BRCA1’s lack of sequence-specific DNA binding, BRCA1 is likely to be recruited to target gene promoters by sequence-specific DNA binding transcription factors^32^. Therefore, we sought to investigate whether BRCA1 binds RRM2 promoter in a direct or indirect manner. Three putative consensus transcriptional response elements: AP-1, Sp1 and E2F1 were identified in the BRCA1-bound RRM2 promoter region^33^ (Fig. 3g). siRNA-mediated knockdown of AP-1 and Sp1 did not significantly impair transcriptional activation of RRM2 promoter, whereas the knockdown of E2F1 reduced RRM2 promoter activation to the same extent as BRCA1. Interestingly, simultaneous knockdown of BRCA1 and E2F1 had no additional impact on RRM2 transcription when compared with either alone (Fig. 4b,c). Consistent with previously published reports^29^,^30^,^34^, ChIP analysis confirmed the binding of E2F1 to RRM2 promoter (Fig. 4d) and its knockdown significantly reduced BRCA1 recruitment to promoter regions amplified by both primer sets (P1 and P2), thereby indicating that BRCA1 binding and transcriptional activation of RRM2 occurs in E2F1-dependent manner (Fig. 4e).

Because BRCA1 has been shown to act directly on replication forks and protect cells from RS-induced DNA damage in other cell types, we next wished to evaluate the extent of RRM2’s contribution to observed RS induction in GBM cells on BRCA1 loss. We found that ectopic expression of RRM2 at least in part rescued the BRCA1 knockdown-associated phenotypes of decreased fork progression speed associated with increased phosphorylation of RPA (assessed by immunoblot analysis), as well as the viability of GBM cells (Fig. 4f–h & Table 3).

### Triapine treatment mimics BRCA1 loss phenotype in GBM

**Figure 5:**
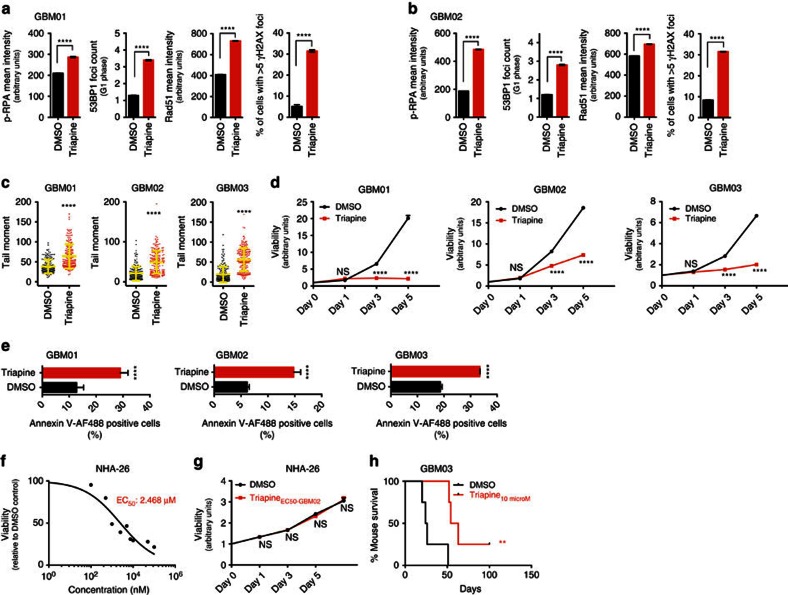
RRM2 inhibition using triapine selectively kills GBM cells and impairs tumour initiating capacity of GBM cells. (**a**) Quantification of p-RPA/ Rad51, 53BP1 and γH2AX in GBM01 cells treated with DMSO or triapine. (**b**) Quantification of p-RPA/Rad51, 53BP1 and γH2AX in GBM02 cells treated with DMSO or triapine. (**c**) Comet assay and tail moment quantification of DSBs in GBM cells treated with DMSO or triapine. (**d**) Viability curves for GBM01, GBM02 and GBM03 cells treated with their respective EC_50_ triapine concentrations over a period of 5 days. (**e**) Quantification of apoptotic cells for GBM01, GBM02 and GBM03 cells treated with their respective EC_50_ triapine concentrations 72 h. (**f**–**g**) Viability-based assessment of EC_50_ triapine concentration in NHA-26 cells and viability curve of NHA-26 cells treated with triapine EC_50-GBM02_. (**h**) Kaplan-Meier survival curve for NMRI nude mice intracranially co-injected with GBM03 cells in DMSO or 10 μM triapine. Statistical significance was calculated by one-way ANOVA, Tukey’s multiple comparisons test (*in vitro* studies) and Log-rank/Mantel-Cox test (*in vivo* study). All data are shown as means ±s.d. and performed as technical triplicates. (*P<0.05, ^**^P<0.005, ^***^P<0.005, ^****^P<0.0001; NS represents non-significance).

The emerging pro-survival role of BRCA1-RRM2 in protecting GBM cells from RS inspired us to search for compounds to target the BRCA1-RRM2 axis and cross the blood-brain barrier (BBB). We came across an RRM2-specific inhibitor triapine (3-aminopyridine-2-carboxaldehydethiosemicarbazone), a drug reportedly effective in two clinical studies on cervical cancer^35^ and currently assessed in several phase I and II trials in other cancer types (https://clinicaltrials.gov/ct2/results?term=triapine). Triapine exhibits neuroprotective effects in a rat model of transient ischaemia^36^. We found that the EC_50_ values for triapine in 3 representative GBM xenografts were in the nanomolar range (GBM01: 518.6 nM; GBM02: 581.2 nM; GBM03: 307.2 nM) (Fig. 4i). Exposure of GBM cells to triapine (EC_50_) *in vitro* caused: (i) decreased replication fork speed (Fig. 4j; Table 4), (ii) elevated p-RPA and Rad51 foci mean intensity in S-phase cells, (iii) increased counts of 53BP1 bodies in G1 cells and (iv) DSBs accumulation (γH2AX quantification and comet assay) (Fig. 5a–c). A single-dose exposure of GBM cells to triapine impaired viability over a period of 5 days and induced apoptosis (Fig. 5d,e). Supportive of likely favourable therapeutic index, astrocytes (NHA-26) were more resistant to triapine compared with GBM lines, with EC_50_ of 2.5 μM and no effect on astrocyte viability when treated with EC_50-GBM02_ or 10 μM triapine (Fig. 5f,g). To further explore the therapeutic potential of triapine, we assessed the effect of direct triapine (10 μM) co-injection with GBM cells on tumour growth *in vivo*. Triapine extended survival of the tumour bearing mice (median survival of 25 days in DMSO control group versus 58.5 days in triapine group; *P*=0.0114, Log-rank (Mantel-Cox) test; Fig. 5h). Triapine is an iron chelator that inhibits the enzymatic activity and the tyrosil radical of the RRM2/p53R2 subunit of RNR. Furthermore, iron chelators can promote release of localized and cytotoxic reactive oxygen species (ROS)^37^. Indeed, we found that triapine treatment enhanced ROS and oxidative DNA lesions (8-oxo-guanine) in all 3 GBM xenograft lines (Supplementary Fig. 4a,b). Triapine not only induced p-RPA, but also increased the PARylation levels, a marker of activated PARP (Supplementary Fig. 4c). We explored the possibility that triapine-induced ROS/8-oxo-guanine sensitizes GBM cell to olaparib. The combined administration of triapine (EC_50-Tria_) and olaparib (EC_50-Ola_ and EC_25-Ola_) was more efficient at reducing GBM cell viability than either drug alone, a combinational effect not observed in NHA-26 or BJ cells used as non-malignant controls (Supplementary Fig. 4d,e). Interestingly, a lower olaparib dose (half dose of EC_50-_olaparib; EC_25-ola_) was equally (GBM03) or more efficient (GBM01 and GBM02) at eradicating GBM cells than full EC_50_ when combined with triapine. This was concordant with our previous work showing that induction of ROS and oxidative DNA lesions renders GBM cells dependent on functional PARP (ref. 38).

### Expression of BRCA1 and RRM2 in malignant gliomas

**Figure 6:**
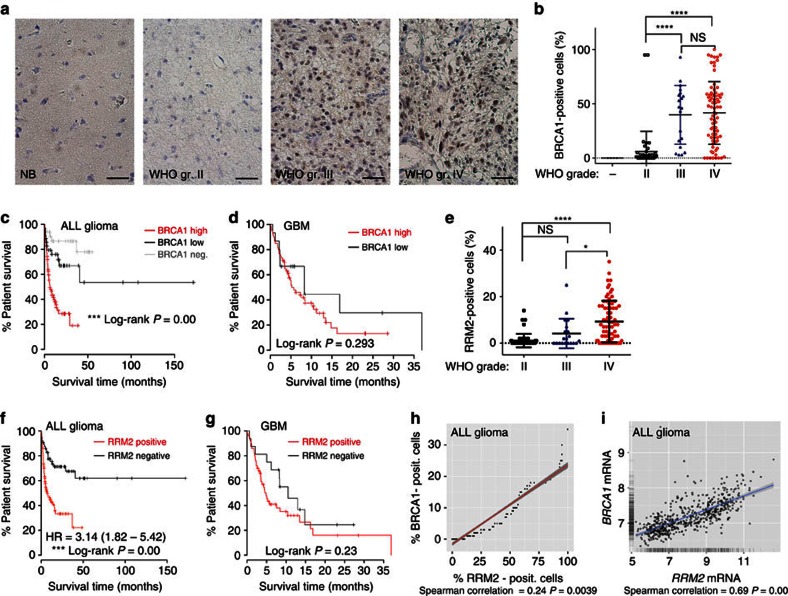
Expression and prognostic value of BRCA1 and RRM2 in malignant gliomas. (**a**) IHC staining of BRCA1 in normal brain (NB), WHO gr. II, III, IV glioma. Shown are representative sections. Scale bar 50 μm. (**b**) Quantification of (**a**) BRCA1 staining. Statistical significance was calculated by one-way ANOVA and Tukey’s multiple comparisons test. (**c**) A survival curve for BRCA1 negative (*n*=32); BRCA1 low (< 14.5% of BRCA1^+^ cells, *n*=35) or BRCA1 high (>14.5% of BRCA1^+^ cells, *n*=78; median survival of 230 days) glioma patients; Log-rank *P* value=0.00. Median survival for BRCA1 negative and low patient is not available as >50% of patients were alive at the end of study. (**d**) A survival curve for BRCA1 high (>14.5% of BRCA1^+^ cells, *n*=60, median survival of 159 days) and BRCA1 low (<14.5% of BRCA1^+^ cells, *n*=15; median survival of 251 days) GBM patients. Log-rank *P*-value=0.293. (**e**) Quantification of RRM2 staining (% of positive cells). Statistical significance was calculated by one-way ANOVA and Tukey’s multiple comparisons test. (**f**) A survival curve for RRM2 negative (*n*=64) and RRM2 positive (*n*=81; median survival of 222 days) glioma patients, where the median RRM2-positivity is 1% and Log-rank *P* value=0.00. Median survival for RRM2 negative patients is not available as more than 50% of patients were alive at the end of study. (**g**) A survival curve for RRM2 positive (*n*=56, median survival of 148 days) and RRM2 negative (*n*=19; median survival of 320 days) GBM patients. Log-rank *P* value=0.23. (**h**) Spearman correlation test confirmed a positive correlation between the percentage of BRCA1 and RRM2-positive cells in human gliomas (our study dataset). (**i**) Spearman correlation test confirmed a positive correlation between BRCA1 and RRM2 mRNA expression in human gliomas (REMBRANDT dataset). (*P<0.05, ^**^P<0.005, ^***^P<0.005, ^****^P<0.0001; NS represents non-significance).

To explore the therapeutic potential of targeting the BRCA1-RRM2 signalling axis in malignant gliomas, we performed immunohistochemistry (IHC) analysis BRCA1 and RRM2 in 145 gliomas (Supplementary Table 2) and 10 non-neoplastic adult brain controls (NB). The mean BRCA1 positivity was increased in WHO grade II gliomas (mean=5.92%) compared with NB (*P*=0.5344, n.s., One-way ANOVA and Tukey’s multiple comparisons test; Fig. 6a,b). Whereas there was no significant difference in the percentage of BRCA1^+^ cells in WHO grade III versus IV gliomas (mean positivity 39.85 and 41.6% of BRCA^+^ cells, respectively), both grade III and IV tumours (high-grade gliomas, HGG) exhibited higher percentage of BRCA1^+^ cells compared with either NB or WHO grade II gliomas (*P*<0.0001, One-way ANOVA and Tukey’s multiple comparisons test; low-grade gliomas, LGG; Fig. 6b). While BRCA1 was undetectable in adult NB (Fig. 6a,b), the cortical plate and its extension into the ammonic plate in the hippocampal anlage were BRCA1 positive in human fetal forebrain (12th week post conception). Parallel IHC staining for Ki67 (proliferative marker) showed that BRCA1 is maximally expressed in non-proliferative (Ki67-negative) regions of the developing human brain (Supplementary Fig. 5a). Similarly, BRCA1 expression was not restricted to proliferating GBM cells *ex vivo* and *in vitro* (Supplementary Fig. 5b). RRM2 positivity was significantly higher in WHO grade IV gliomas (mean=9.3%) than in WHO grade III (mean=4.15%; *P*=0.0134, One-way ANOVA and Tukey’s multiple comparisons test) and grade II (mean=1.1%; *P*<0.0001, One-way ANOVA and Tukey’s multiple comparisons test), whereas there was no significant difference in mean positivity between WHO grade II and III tumours (Fig. 6e). Next, we validated these findings by ‘*in silico*’ analysis of the publically available REMBRANDT glioma dataset (GlioVis web application). Both, *BRCA1* and *RRM2* mRNA levels were significantly higher in HGG (WHO grade III+IV) compared with LGG (WHO grade II), whereas the *RRM2* expression was also significantly higher in WHO grade IV tumours compared with WHO grade III (Supplementary Fig. 5c), thereby supporting our findings above.

### Prognostic value of BRCA1 and RRM2 in malignant gliomas

To further correlate the fraction of immunohistochemically BRCA1^+^ and RRM2^+^ cells and overall survival, we analysed their prognostic significance using the Cox regression method. As shown in Fig. 6c, BRCA1 high patients (>14.5%, median survival=230 days, were 14.5% represents median BRCA1 positivity in our cohort) had significantly shorter overall survival than BRCA1 low patients (<14.5%, median survival not yet available as over 50% of patients were alive at the end of this study) or patients negative for BRCA1 (BRCA1 negat.; median survival also unavailable as over 50% of patients were alive, Log-rank *P*=0.00). The median RRM2 positivity (% of RRM2^+^ cells) in our cohort was 1% and Cox regression analysis showed that glioma patients negative for RRM2 (RRM2 negat., median survival not reached) had longer survival times than RRM2 positive patients (RRM2 positive; median survival=222 days, Log-rank *P*=0.00; Fig. 6f). The univariate Cox regression analysis revealed BRCA1 and RRM2 are negative prognostic factors informing worsen patient survival (HR=7.72, *P*=0.00, 95% CI 2.98–19.97 for BRCA1 high; HR=3.14, 0=0.00, 95% CI 1.82–5.42 for RRM2; Supplementary Table 3). According to multivariate analysis (Supplementary Table 3) both, BRCA1 and RRM2 positivity, correlate with WHO malignancy degree and patient age, but are independent of proliferative index (% of Ki67^+^ cells). Cox regression analysis of WHO grade IV (GBM) patients divided into BRCA1 low (<14.5%) and BRCA1 high (>14.5%) or RRM2 negative and positive groups showed a trend toward worse survival of BRCA1 high and RRM2 positive patients (251 days for BRCA1 low versus 159 days for BRCA1 high and 320 days for RRM2 negat. versus 148 for RRM2 posit.; Fig. 6d,g, respectively).

Next, we sought to determine whether there is a correlation between BRCA1 and RRM2 expression in our clinical glioma cohort. Spearman correlation analysis (SCA) for association revealed a positive correlation between the percentage of BRCA1^+^ and RRM2^+^ cells in our glioma cohort (correlation coefficient=0.24; *P*=0.0039; Fig. 6h). Furthermore, we validated these findings by ‘*in silico*’ analysis of the publically available REMBRANDT glioma dataset (GlioVis web application), which confirmed the positive correlation between *BRCA1* and *RRM2* mRNA expression (SCA; *P*=0.00; CI 0.44–0.57; Fig. 6i). No correlation between *BRCA1* and *RRM2* was found in normal brain controls (SCA; *P*=0.8; CI −0.57–0.68).

The validation study using the REMBRANDT glioma dataset (ALL gliomas and GBM) confirmed the prognostic significance of *BRCA1* and *RRM2* mRNA expression in malignant gliomas. *BRCA1 high* patients showed significantly shortened survival compared with *BRCA1 low* patients when either ALL gliomas or just GBM patients were analysed (Supplementary Fig. 5d,e). Similarly, high *RRM2* expression (*RRM2 high*) analysis was associated with worsen survival of glioma, in general, and GBM patients, in particular (Supplementary Fig. 5f,g). The correlation between BRCA1 or RRM2 positivity and GBM patient survival was surprising as our results from multi-variate analysis showed strong association of both factors with WHO grade. Hence, we sought to investigate whether this was attributable to differences in BRCA1 and RRM2 positivity in GBM molecular subtypes^39^. This was indeed the case, as shown in Supplementary Fig. 6a, both *BRCA1* and *RRM2* mRNA expression was markedly higher in classical GBMs (compared with mesenchymal and proneural subtype), which was reflected in the worsen survival of *BRCA1 high* and *RRM2 high* patients with classical GBM tumours (Supplementary Fig. 6b,c).

Altogether, our data show that BRCA1 loss causes impeded replication fork speed aggravating the endogenous RS and accumulation of DSBs in GBM cells. Transition through the cell cycle with enhanced amounts of aberrant replication intermediates and/or DSBs (if left unrepaired) may result in aberrant mitoses and cell death. Our data show that the abrogation of BRCA1 function impairs tumorigenicity of GBM cells, implying a novel stress-support, pro-tumorigenic role for BRCA1. Importantly, our present results provide the first evidence of BRCA1 and its down-stream effector RRM2 as negative prognostic factors for glioma patient survival. Last but not least, our experiments with triapine suggest a potential new therapeutic avenue for highly aggressive GBM.

## Discussion

Error-free and timely regulation of cell cycle progression is crucial for genome integrity maintenance. Cells are particularly sensitive during S phase when DNA damage causes replication fork stalling or collapse, collectively referred to as replication stress, one of the emerging hallmarks of cancer^40^. If not restarted in a timely manner, stalled forks collapse into DSBs, potentially yielding deleterious chromosomal rearrangements^4^. To cope with such problems, cells evolved a complex monitoring system called S-phase checkpoint, which becomes activated in response to stalled forks and DNA damage in order to trigger appropriate cellular responses. The tumour suppressor BRCA1 plays a critical role in maintaining genomic stability in general, and RS in particular^8^,^41^. Preclinical and retrospective clinical data suggest that BRCA1 mutation may provide a novel predictive marker of response to chemotherapy^42^. Our present findings provide the first clear evidence that BRCA1 positivity increases with increasing degree of malignancy in human gliomas and serves as a negative prognostic factor for patient survival (Fig. 6, Supplementary Fig. 5).

Numerous reports have demonstrated that the wild type BRCA1 loss impairs the growth of several cancers (breast, ovarian, lung, prostate and colon)^14^,^16^,^43^,^44^,^45^,^46^,^47^. In addition, the work by Holt *et al.* has shown that over-expression of BRCA1 causes growth retardation in breast and ovarian cell lines^48^. In our studies, BRCA1 loss resulted in massive RS-induced DNA damage associated with apoptosis and impaired GBM growth. Our data indicate, that this phenotype is at least in part attributable to down-regulation of RRM2 upon BRCA1 knockdown (Figs 1, 2, 3). Even though the reduction in RRM2 levels was moderate, we believe it was sufficient to induce RS as evidenced by a number of assays. This is consistent with previous reports showing that even a moderate increase RRM2 activity and so dNTP pools protects cells from RS by promoting replication fork progression^49^,^50^. To rule out that the drop in RRM2 levels was not simply a cell cycle effect, because BRCA1 knockdown arrested cells in S-phase in which RRM2 levels are known to be the highest^51^, we have performed cell cycle analysis with nocodazole arrest (Fig. 1e). Despite marked accumulation of cells in S phase, these were still progressing from S to G2/M phase. In addition, the analysis of RRM2 expression in individual cell cycle (G1-S-G2/M) phases confirmed that after BRCA1 knockdown, RRM2 levels decreased in all cell cycle phases (Fig. 3f). To evaluate whether this phenotype is unique to GBM or applies to other cell types, we performed BRCA1 knockdown in 4 additional cancer and 3 non-malignant cell lines. Our experiments showed no major impact on growth characteristics of human ovarian (OVCAR5) and breast cancer (Cal51) cell lines upon BRCA1 knockdown. Prostate (PC3) and cervical carcinoma (HELA) cell lines experienced lowered viability and cell cycle arrest at G2/M after BRCA1 knockdown, but continued growing (Supplementary Fig. 2a). Since the inhibition of endogenous BRCA1 expression in mouse fibroblasts led to their malignant transformation and increased apoptotic rates in neural progenitor cells^47^,^52^, we also evaluated the effect of BRCA1 knockdown in foreskin human fibroblasts (BJ) and normal human astrocytes (NHA). Both cell types responded by decreased viability and cell cycle arrest (Supplementary Fig. 1a,c). Importantly, none of these cells (non-malignant or non-GBM cancers) exhibited either reduced RRM2 protein levels or S-phase arrest, thereby indicating a unique mechanism used by GBM cells for protection to supra-physiological levels of RS. Interestingly, we have observed markedly increased baseline levels of RPA phosphorylation and oxidative DNA lesions (8-oxo-guanine) in GBM cells in comparison to above evaluated non-GBM cancer and non-malignant cell lines (Supplementary Fig. 4f), which allow us to speculate that our results reflect differential starting levels of endogenous RS and hence selective raise of RS to intolerably high levels in only the GBM models, or some more fundamental differences between RS responses in GBM versus epithelial cancers, a task which remains to be investigated in future studies.

Recent evidence identified a link between BRCA1 and DNA repair through transcriptional regulation of the DDR (ref. 32). BRCA1 stabilizes p53, thereby directing a selective transcriptional response towards cell-cycle arrest and DNA repair^53^. Our biochemical data revealed a novel BRCA1 transcriptional target—*RRM2.* Further investigations suggested that the BRCA1-regulated *RRM2* expression in GBM cells was dependent on E2F1, a transcriptional factor previously reported as transcriptional activator of *RRM2* (refs 30, 33) (Fig. 4c,e). A number of transcription factors has been shown to trans-activate the *RRM2* gene, including E2F1, Sp1, AP-2, CTF-1/NF1- and NF-Y/CAATA, some of which have been shown to directly interact with BRCA1 at regulatory DNA sequences. Thus, the regulatory interplay of BRCA1 and E2F1 in the control of RRM2 transcription uncovered in our present study, will be followed by further experiments to understand the specific mechanism and how BRCA1 interacts with E2F1 and possibly additional proteins at the RRM2 gene promoter.

Even though we confirmed BRCA1 binding to RRM2 promoter in NHA and BJ cells, BRCA1 loss was not associated with reduction in RRM2 protein levels in these cells (Supplementary Fig. 1d), thereby indicating that this regulatory mechanism is unique to GBM cells.

Ectopic expression of RRM2 rescued the BRCA1-depletion phenotype (RS/viability), thereby underscoring the importance of this newly identified BRCA1 function. Additionally, RRM2 expression positively correlated with that of BRCA1 and both the percentage of BRCA1^+^ and RRM2^+^ cells correlated negatively with glioma patient survival (Fig. 6c,e,f,h; Supplementary Fig. 5c,e), thus making RRM2 an attractive candidate for therapeutic targeting in GBM. Supraphysiological levels of RRM2 can lead to genomic instability and tumorigenesis due to imbalanced dNTP pools, underlining its ‘proto-oncogenic’ role^54^,^55^. Here, chemical inhibition of RRM2 with triapine undermined viability and tumour initiating capacity of GBM cells due to increased replication and oxidative stress. Importantly, NHA-26 cells were resistant to doses of triapine used to target GBM cells and EC_50-NHA26_ was significantly higher, consistent with reports suggesting the use of triapine as a neuroprotectant^36^. The cellular consequences of replication and oxidative stress depend on the extent of DNA damage and the capacity to activate DDR and repair the damage. Our previous work and that of others showed that malignant gliomas display aberrant activation of the DDR, due to ongoing RS and the presence of oxidative DNA lesions^22^,^26^,^56^. In the present study, we show that BRCA1 provides GBM cells with an additional fork protection mechanism, distinct from the pathways reported so far. BRCA1 loss in GBM cells resulted in reduced replication fork speed and impaired recovery of HU-induced fork stalling (Fig. 3a,b). To prevent DSBs during replication, stalled forks need to restart before collapsing. Impaired fork recovery after BRCA1 knockdown and S-phase arrest coincided with DDR signalling and increased frequency of p-RPA^+^/γH2AX^+^ GBM cells altogether indicating the conversion of stalled forks into DSBs. In response to replication inhibitors such as HU, PARP-1 reportedly interacts with MRE11 and promotes MRE11 foci formation, ssDNA generation and replication fork restart^3^,^57^. We now show that impairment of replication fork restart may potentiate effects of olaparib on triapine treated GBM cells (Supplementary Fig. 4), a phenomenon to be explored in future studies.

Given that neither BRCA1 nor RRM2 is expressed in normal adult brain, their function as a ‘RS’-support pathway provides a promising rationale for clinical application of triapine, possibly combined with PARP inhibitors or standard-of-care genotoxic treatments. We believe that apart from the discovery of the BRCA1-RRM2 interplay as a novel example of non-oncogene addiction^58^ in GBM, our results also offer testable predictive biomarkers (BRCA1/RRM2), and therefore may inspire further work to validate clinical relevance of our present findings.

## Methods

### Patients

We performed a retrospective study of 75 patients with a diagnosis of glioblastoma WHO grade IV (GBM, 66 primary and 9 secondary), 20 patients with a diagnosis of anaplastic astrocytoma WHO grade III and 50 patients with a diagnosis of astrocytoma WHO grade II. Informed written consent was obtained for all patients, which were identified through the database of the University Hospital Olomouc and Na Homolce Hospital Prague (Supplementary Table 2). IRB approval was obtained for tissue collection (Hospital Na Homolce Institutional Ethics Committee protocol #3.3.2016/1).

All patients in the study underwent brain biopsy or tumour resection between years 2011 and 2014 at the Departments of Neurosurgery (University Hospital Olomouc or Na Homolce Hospital Prague, CZ). Subsequently, the material was processed at the Department of Clinical and Molecular Pathology (University Hospital Olomouc, CZ) or at the Department of Pathology (Na Homolce Hospital Prague, CZ). It was examined by at least two histo-pathologists in order to make the diagnosis according to the current WHO classification^59^.

### Cell isolation and culturing

Xenografted glioblastoma (GBM) cells GBM01 (IN1123), GBM02 (IN84) were a generous gift from Dr I. Nakano (The Ohio State University, USA). Non-neoplastic brain cells (NB34, derived from epilepsy patient) and GBM03 (4121) cells were provided by Dr JN Rich (Cleveland Clinic, USA). GBM cells were derived from specimens of neurosurgical resection directly from patients in accordance with an Ohio State University or Cleveland Clinic Institutional Review Board-approved protocol in which informed consent was obtained by the tumour bank which provided deidentified excess tissue to the laboratory. Low grade glioma (LGG-1636 and LGG-67, WHO grade II) cells were derived from neurosurgical resections at Copenhagen University Hospital (Department of Neurosurgery) directly from patients in accordance with the Danish Ethical Committee guidelines (Protocol Number H-3-2009-136) including an informed consent acquired 24 h prior surgery. Normal Human Astrocytes were purchased from 3H biomedical and cultured in Astrocyte Basal Medium supplemented with SingleQuots, as recommended by the manufacturer. Whole Brain Extract used for immunoblot analysis was purchased from Novus Biologicals. No-GBM cancer cancer cell lines: PC3, HELA, Cal51 and OVCAR5 (ATCC), were used as tissue specific controls and cultured as recommended by the manufacturer. GBM cells were maintained through subcutaneous xenografting in the flanks of BALB/c (nu/nu) mice. Tumours were dissected out and dissociated using papain dissociation system (Worthington Biochemical). Dissociated cells (max culture time 24 h post dissection from mice) were cultured in Neurobasal A media supplemented with B27 Supplement Minus vitamin A (Invitrogen), epidermal growth factor and basic fibroblast growth factor (10 ng ml^−1^, Invitrogen). Cells were cultured at 37 °C in an atmosphere of 5% CO_2_. For cell counting before each experiment, single-cell suspension was prepared using TrypLE (Invitrogen).

### Lentiviral shRNA particle preparation and cell transduction

Lentiviral shRNA particles (Sigma-Aldrich, non-targeting shRNA control plasmid SHCO16-1EA (shCTRL), TRCN0000244985 (shBRCA1-2), TRCN0000244987 (shBRCA1-4), TRCN0000244988 (shBRCA1-5) were prepared as described previously^60^ and equal amounts of virus were used for infection three days post infection cells were collected and used downstream assays. The level of BRCA1 knockdown was assessed by immunoblotting.

### Wet reverse pDNA transfection

GBM01 cells stably overexpressing RRM2 were generated by wet reverse transfection using the X-tremeGENE HP DNA Transfection Reagent (Roche, cat. no. 06 366 244 001). pcDNA3 RRM2 was a gift from Edward Whang (Addgene plasmid #13796) and pcDNA3.1 empty vector was used as control (Invitrogen). Protocol in brief, 6 μl transfection reagent and 2 μg plasmid DNA of interest was incubated for 15 min at room temperature and then spread on the bottom of a well in a 6 well plate. 200,000 GBM01 cells were seeded on top of the transfection solution in 2 ml complete medium and cultured overnight before plating in selection medium.

### siRNA transfection

siRNA knockdown was performed by wet-reverse transfection using HiPerFect transfection reagent according to manufacturer’s protocol (Qiagen, HiPerFect transfection reagent, cat no 301704). In brief, siRNA (50 nM from Ambion: siRNA-Sp1 ID: 116546, siRNA-AP1/JUN ID: 145018, siRNA-AP-1/Fos ID: 115631; and Sigma: siRBA-E2F1 cat no. EHU070981) and HiPerFect Reagent were mixed and spotted into wells. After complex formation (15 min incubation), cells were plated on the mixture in their respective media and incubated for 72 h before assessing the efficiency of knock down. See Supplementary Fig. 3b for knockdown efficiency validation using immunoblot analysis of E2F1, Sp1 and AP-1 expression.

### Luciferase reporter assay

The luciferase reporter assay was performed with the use of a Dual-Glo Luciferase Assay System according to manufacturer’s instructions (Promega). In brief, cells were transfected with pGL3-RRM2-firefly construct (a generous gift from Dr Wang, Dept. of Mol. Cardiology, Lerner Research Institute/NB50, Cleveland, OH, USA) using PEI transfection reagent (PEI, Polyethylenimine, Polysciences, Inc.). In each experiment, a control plasmid expressing *Renilla* luciferase (at a ratio 1:2) was co-transfected. Fourty eight hours post transfection, cells were harvested, and firefly luciferase reporter and *Renilla* control luciferase activities were measured. Firefly luciferase reporter activity was expressed as a fold change following normalization to *Renilla* luciferase activity.

### Chromatin immunoprecipitation

Cells (10^8^) were harvested, washed with PBS, resuspended in cold PBS, and incubated in PBS with 1% formaldehyde for 10 min at RT. Crosslinking reaction was stopped by addition of glycine to 0.125 M. Cells were washed with PBS, resuspended in ChIP lysis buffer (10 mM EDTA, 50 mM Tris pH 8.1, 0.5% SDS), and sonicated to 300–500 bp average DNA fragment size. Debris was removed by centrifugation (13,200*g* for 10 min at 4 °C) and the supernatant diluted 10-fold in ChIP binding buffer (150 mM NaCl, 20 mM Tris–HCl (pH=7.5), 0.1% Nonidet NP-40, protease inhibitors (EDTA-free, Roche), and phosphatase inhibitors (PhosSTOP, Roche)) and incubated with anti-BRCA1 (or E2F1; Abcam, ab4070) or isotype-matched rabbit anti-IgG control (negative control) or anti-Histone H3K9ac (Abcam, ab4441, positive control—see Supplementary Fig. 3a) overnight at 4 °C with agitation. The next morning, 50 μl protein A/G sepharorse beads (blocked in ChIP binding buffer containing single stranded herring sperm DNA at 0.1 μg μl^−1^)) were added for 1 h. Beads were washed six times (ChIP binding buffer+0.1% SDS). Crosslinked protein–DNA complexes were eluted for 15 min at RT with elution buffer (100 mM NaHCO_3_, 1% SDS) and incubated in NaCl at 100 mM overnight at 65 °C to reverse crosslinks. DNA was purified using QIAGEN QIAquick PCR kit and used as template for q-PCR quantification on the Applied Biosystems 7500 Fast Real Time PCR System. Calculations were carried out using the per cent input method according to guidelines from Thermo Fisher Scientific guidelines (https://www.thermofisher.com/us/en/home/life-science/epigenetics-noncoding-rna-research/chromatin-remodeling/chromatin-immunoprecipitation-chip/chip-analysis.html) to include normalization for both background levels and input chromatin going into the ChIP. First, the input is adjusted to 100%. Since the starting input fraction is 1%, a dilution factor of 100 (corresponding to 6,644 cycles) is subtracted from the Ct value of the diluted input. Next, the per cent input is calculated using the equation: 100 × 2ˆ(adjusted input- average triplicated Ct (IP)). RRM2 primer sets P1 and P2 (for sequence see Fig. 3g) were designed based on human RRM2 promoter sequence with GenBank accession number: AY032750 (ref. 33).

### RNA extraction and Real-Time PCR

RNA from GBM01 and GBM02 (transduced with lentiviral shCTRL or shBRCA1-2 or shBRCA1-4 particles) was extracted using an RNeasy Plus Mini kit (QIAGEN #74134) according to the manufacturer’s instructions. RNA concentrations were measured with a NanoDrop spectrophotometer and samples were stored in −80 °C freezer. cDNA was synthesized from total RNA using the High Capacity cDNA reverse transcription kit (Applied Biosystems). Real-time PCR (quantitative PCR) was performed using Fast SYBR Green Master mix (Applied Biosystems) according to manufacturer’s instructions. Amplification was performed in the Applied Biosystems 7500 Fast Real Time PCR System. Primer sequences: BRCA1 (forward primer: ACTGCAGCCAGCCACAGGTA; reverse primer: TAGCCAGGACAGTAGAAGGA), RRM2 (forward primer: TTACATAAAAGATCCCAAAGAAAGG, reverse primer: AGCCTCTTTGTCCCCAATC, β-actin forward CCAACCGCGAGAAGATGA; reverse CCAGAGGCGTACAGGGATAG) β-actin was used as an internal control and the ΔΔCT method was used to calculate changes in fold expression.

### Immunohistochemistry

Ten-micrometre thick sections were prepared from formalin-fixed, parafin-embedded neoplastic tissue. The sections were pretreated in microwave oven to retrieve the antigen. The endogenous peroxidase activity was blocked using 6% H_2_O_2_. The sections were then incubated for 1 h with mouse monoclonal primary antibody against BRCA1 (1:200, IHC-00278, Bethyl Laboratories, Montgomery, TX), RRM2 (1:500, Abcam, ab57653) or Ki67 (DAKO) and followed with Dako EnVision+ Dual Link System-HRP secondary antibody (DAKO, Glostrup, Denmark) incubation for 1 h at room temperature. The immunoreactivity was visualized by liquid DAB+ substrate-chromogen system (DAKO, Glostrup, Denmark). Finally, slides were washed under running water, dehydrated through graded ethanol and mounted. The nuclei were counterstained with hematoxylin. Immunostaining on each slide was scored by an experienced pathologist, to examine the percentage of BRCA1/RRM2 positive tumour cells. At least 400 GBM cells were counted in 10 large, random microscopic fields per section. Patients were divided into BRCA1 high and BRCA1 low groups based on the calculated median positivity of 14.5% BRCA1-positive cells. Patients were divided into RRM2 positive (>0%) and RRM2 negative groups based on the median positivity (=1%).

### Immunofluorescence and microscopy

Immunofluorescence staining of Rad51 (Abcam, ab213, 1:250), γ-H2AX ser139 (Millipore, 05-636, 1:1,000), pRPA (Thr21) (Abcam, ab61065, 1:2,000), BRCA1 (Bethyl laboratories, IHC-00278, 1:500), PCNA (Immuno Concepts, 2037, 1:100), 53BP1 (Millipore, MAB3802, 1:700) were performed as described previously^56^. GBM cells were grown on GelTrex (Invitrogen)-coated coverslips and treated with shRNA-mediated knock down over a 72 h period. NHA cells were grown to appropriate confluence and treated with 2 mM HU or DMSO vehicle for 2 h. Subsequently, cells were fixed with 4% PFA and immunostained with the indicated primary antibody. Nuclei were counterstained with DAPI (Sigma-Aldrich). Imaging was performed using LSM 700 META/imager.Z1 (plan-apochromat 63 × /1.40 oil DIC M27 objective, Carl Zeiss, Inc.). Confocal images were acquired with equal settings and processed with Zen 2008 software (Carl Zeiss, Inc.). For quantification, 100 non-overlapping images were acquired for each condition using the ScanˆR screening station (Olympus). At least 1,000 cells were scored and processed using ScanˆR Analysis software (Olympus).

### Immunoblot analyses

Whole-cell extracts were separated by 6, 8 or 15% SDS–PAGE and transferred to nitrocellulose membranes (Biorad) using wet electroblotting system (Bio-Rad laboratories). The membranes were blocked using 5% (w/v) dry milk in PBS-Tween-20 (0.5% vol/vol) and probed with appropriate primary antibodies (Supplementary Table 4). The antibody against α-tubulin was used as loading control. ECL detection system was used according to manufacturer’s instructions (GE Healthcare). Uncropped scans of the most important blots are provided in Supplementary Figs 7 and 8.

### Cell viability

Dissociated single cells were plated into a 96-well plate at 3,000 cells/well in triplicates. Next day, vehicle (DMSO) and triapine (EC_50_) were added and cell viability was measured over a period of up to 7 days using CellTiter-Glo Luminescent Cell Viability Assay (Promega) and results were calculated as relative fold change in ATP with each group internally normalized to the respective vehicle control. For BRCA1 shRNA knockdown experiments, cells were plated three days post lentiviral transduction. For each cell line (GBMs and NHA), the appropriate EC_50_ concentration of a drug was calculated and used for all further inhibitor experiments.

### Small molecule inhibitor and EC_50_ calculations

The small molecule inhibitor triapine (Selleck Chemicals S7470) and PARP inhibitor olaparib (Selleck Chemicals, AZD2281) were dissolved in DMSO at 20 mg ml^−1^ and 5 mg ml^−1^, respectively. DMSO at a final percentage equivalent to that of the drug stock solution served as vehicle control for all studies. For each cell line, the appropriate EC_50_ concentration of triapine was calculated and used for all further experiments. For EC_50_ calculation, acutely dissociated single cells were plated (3,000 cells per well in a 96-well in triplicate). Next day, vehicle or drug was added and cell viability was measured using CellTiter-Glo Luminiscent Cell Viability Assay (Promega) 72 h later. EC_50_ was calculated using non-linear regression in GraphPad Prism Software.

### DNA fibre assay

GBM cell cultures (transduced with respective shRNA or treated with triapine inhibitor) were pulse-labelled with 25 μM of CldU for 20 min, followed by the change of media and a second pulse of 250 μM of IdU for 20 min. For the fork recovery assay cells were treated with 25 μM of CldU for 20 min followed by exposure to 2 mM hydroxyurea for 4 h or left untreated. Labelled cells were harvested, lysed and DNA fibre spreads prepared as described previously (Maya-Mendoza *et al.*, 2012). Further to detect CldU a rat anti-BrdU antibody (OBT0030, Serotec, 1:500) was used and for IdU detection a mouse anti-BrdU antibody (347580, Becton Dickinson, 1:500). Antibodies for secondary detection used were anti-rat AlexaFluor564 and anti-mouse AlexaFluor488, respectively with dilution 1:500. Images of well spread DNA fibres were taken using the LSM700 Zeiss microscope with the × 63 oil immersion objective. Double-labelled replication forks were analysed manually using ZEN software. Presented results are from replicates of 2–3 independent experiments. The rate for nascent tract replication was estimated using the conversion of 2.59 kb μm^−1^ (ref. 61).

### Flow cytometry and cell cycle analysis

Flow cytometry was performed using FACS Verse Cell Sorter (BD Biosciences) and analysed using FlowJo software. For Annexin-V staining, cells were labelled with Alexa Fluor 488-conjugated Annexin-V for 15 min in Annexin V-binding buffer according to the manufacturer’s instructions (Invitrogen). Click-it EdU kit (Invitrogen) was employed to assess the cell cycle profile and quantify the percentage of S-phase cells. Briefly, cells were incubated with EdU (5-ethynyl-2′-deoxyuridine) at a concentration of 10 μM for 20 min and subsequently, detection of EdU was performed according to manufacturer’s instructions (Invitrogen). To allow for quantification of mitotic cells and RRM2- or p-RPA/γH2AX- or PCNA-γH2AX-positive cells, cells were further labelled with anti-H3^Ser10^, anti-RRM2, anti-p-RPA+anti-γH2AX or anti-PCNA+γH2AX antibodies, respectively. For G2/M arrest experiment, cells were treated with nocodazole (0.04 μg ml^−1^, Sigma-Aldrich) for 12 h prior the analysis. In all analyses, DNA was counterstained with Hoechst 33342 dye (Invitrogen). Measurement of ROS (H2-DCF probe, Invitrogen) and 8-oxo-2′-deoxyguanosine (using anti-8-oxo-2′-deoxyguanosine antibody followed by Alexa Fluor 488-conjugated secondary antibody) levels were measured as described previously^22^. Samples were acquired using FACSverse (BD Biosciences) and FlowJo software was used for data analysis.

### Alkaline comet assay

Single-cell gel electrophoresis under alkaline conditions was performed as described previously^62^. Briefly, cells were harvested into a single-cell suspension in Neurobasal A media, mixed with 0.5% low-melting-point agarose (Gibco) in PBS and spread on a microscope slide pre-coated with 1% normal-melting-point agarose (Invitrogen). Cells were lysed overnight (2.5 M NaCl, 100 mM EDTA, 10 mM Tris, 1% Triton X-100) and subsequently rinsed in neutralization buffer (0.4 M Tris–HCl, pH 7.4). Electrophoresis was carried out in alkaline electrophoresis solution at 25 V for 25 min and fixed in 96% ethanol. DNA was stained using SYBR Green I (Molecular Probes), visualized using fluorescence microscopy (Axiovert 200 M, Carl Zeiss) and analysed using Comet Assay IV software. The mean of at least 200 olive tail moments was calculated. Olive tail moment (OTM) is the product of the amount of DNA in the tail and the mean distance of migration in the tail.

### Animals and *in vivo* tumour formation studies

All animal studies described were approved by the Danish Regulations for Animal Welfare (Protocol Number 2012-15-2934-00636). For *in vivo* BRCA1 shRNA tumour formation studies, 50,000 viable cells (trypan blue method was employed to exclude dead-cell prior counting using Countess automated cell counter, Life Technologies) were stereotactically implanted into the right frontal lobe of Balb/c nu/nu mice (female, 8 weeks). For triapine inhibitor study, GBM03 cells were treated with vehicle or triapine (10 μM). Twenty four hour later 20,000 viable cells were stereotactically implanted into the right frontal lobes of NMRInu-F mice (female, 8 weeks). Mice were monitored daily for neurological impairment and weight loss, at which point they were sacrificed.

### Retrospective analysis of *BRCA1*/*RRM2* expression in gliomas

Correlations between glioma grade, molecular GBM subtype (classical, mesenchymal and proneural), patient survival and *BRCA1* or *RRM2* mRNA expression were determined through analysis of the National Cancer Institute's Repository for Molecular Brain Neoplasia Data (REMBRANDT), which is available through GlioVis (http://gliovis.bioinfo.cnio.es/; Bowman R. and Squatrito M. manuscript in preparation). High and low groups were defined as above and below the mean, respectively.

### Statistical analysis

All the data are represented as mean±s.d. and all the *n* values represent the biological replicates. A Student’s *t* test by log-rank or one-way ANOVA supplemented with Turkey’s multiple comparisons test were used. All the statistical analyses of *in vitro* and mouse experiments were done by using GraphPad Prism 6 software (GraphPad Software, Inc.). For animal survival studies, Kaplan-Meier curves were generated using GraphPad Prism 6 software and log rank analysis performed. Animals were randomly assigned to treatment group. For analysis of the patient cohort the statistical analyses were done by using R (R Development Core team). We applied Cox Proportional Hazard Regression to study the effect of BRCA on survival, both unadjusted and adjusted for confounders age, gender, WHO. All tests were likelihood ratio tests. (*P<0.05, **P<0.005, ***P<0.005; NS represents non-significance).

### Data availability

All data generated during this study are included in this published article (and its supplementary information files) or available from the author upon request. The data sets (REMBRANDT) analysed during the current study are available in the GlioVis repository (http://gliovis.bioinfo.cnio.es/).

## Additional information

**How to cite this article:** Rasmussen, R. D. *et al.* BRCA1-regulated RRM2 expression protects glioblastoma cells from endogenous replication stress and promotes tumorigenicity. *Nat. Commun.* **7,** 13398 doi: 10.1038/ncomms13398 (2016).

**Publisher's note:** Springer Nature remains neutral with regard to jurisdictional claims in published maps and institutional affiliations.

## Supplementary Material

Supplementary Information Supplementary Figures 1-8, Supplementary Tables 1-4

## Figures and Tables

**Table 1:** Statistics and comparison of data in Fig. 3a.

	**Mean fork speed (kb per min)**	**s.d.**	**s.e.m.**	***n***
GBM01				
shCTRL	0.6541	0.2159	0.0093	538
shBRCA1-2	0.4195	0.1613	0.0070	527
shBRCA1-4	0.4991	0.2027	0.009	501
				
GBM02				
shCTRL	0.5733	0.1831	0.0078	546
shBRCA1-2	0.3536	0.1097	0.0045	589
shBRCA1-4	0.3839	0.1398	0.0058	589

Statistical significance was calculated by Student’s *t* test: GBM01 cells: shCTRL versus shBRCA1-2 (****P<0.0001); shCTRL versus shBRCA1-4 (****P<0.0001). GBM02 cells: shCTRL versus shBRCA1-2 (****P<0.0001); shCTRL versus shBRCA1-4 (****P<0.0001).

**Table 2:** Statistics and comparison of data in Fig. 3b.

**GBM01**	**shCTRL**	**shBRCA1-2**	**shBRCA1-4**
Mean	0.5604	0.5193	0.4834
s.d.	0.0157	0.0348	0.0274
s.e.m.	0.0007	0.0016	0.0012

Statistical significance was calculated by Student’s *t* test: Fold comparison (2 mM HU/H_2_O) for shCTRL versus shBRCA1-2 (****P<0.0001) and fold comparison for shCTRL versus shBRCA1-4 (****P<0.0001).

**Table 3:** Statistics and comparison of data in Fig. 4f.

**GBM01**	**Mean fork speed (kb per min)**	**s.d.**	**s.e.m.**	***n***
pcDNA/shCTRL	0.6914	0.309	0.0138	500
pcDNA/shBRCA1-2	0.4033	0.1829	0.0081	500
pcDNA/shBRCA1-4	0.504	0.2305	0.0131	500
pcDNA-RRM2/shCTRL	0.5268	0.2186	0.0098	500
pcDNA-RRM2/shBRCA1-2	0.7508	0.2781	0.0124	500
pcDNA-RRM2/shBRCA1-4	0.975	0.3334	0.0149	500

Statistical significance was calculated by Student’s *t* test: pcDNA/shBRCA1-2 versus pcDNA-RRM2/shBRCA1-2 (****P<0.0001); pcDNA/shBRCA1-4 versus pcDNA-RRM2/shBRCA1-4 (****P<0.0001).

**Table 4:** Statistics and comparison of data in Fig. 4j.

	**Mean fork speed (kb per min)**	**s.d.**	**s.e.m.**	***n***
GBM01				
DMSO	0.4764	0.1722	0.0077	500
triapine	0.3595	0.1507	0.0067	500
				
GBM02				
DMSO	0.5256	0.1939	0.0087	500
triapine	0.3385	0.0909	0.0041	500

Statistical significance was calculated by Student’s *t* test: GBM01 cells: DMSO versus triapine (****P<0.0001); GBM02 cells: DMSO versus triapine (****P<0.0001).
