# Author Correction: BRCA1-regulated RRM2 expression protects glioblastoma cells from endogenous replication stress and promotes tumorigenicity

**DOI:** 10.1038/s41467-018-07892-6

**Published:** 2018-12-19

**Authors:** Rikke D. Rasmussen, Madhavsai K. Gajjar, Lucie Tuckova, Kamilla E. Jensen, Apolinar Maya-Mendoza, Camilla B. Holst, Kjeld Møllgård, Jane S. Rasmussen, Jannick Brennum, Jiri Bartek, Martin Syrucek, Eva Sedlakova, Klaus K. Andersen, Marie H. Frederiksen, Jiri Bartek, Petra Hamerlik

**Affiliations:** 1 Brain Tumor Biology, Danish Cancer Society Research Center, Strandboulevarden 49, Copenhagen, DK-2100 Denmark; 2 Department of Clinical and Molecular Pathology, Faculty of Medicine and Dentistry, Palacky University and University Hospital Olomouc, Hnevotinska 3, Olomouc, 77515 Czech Republic; 3 Genome Integrity Unit, Danish Cancer Society Research Center, Strandboulevarden 49, Copenhagen, DK-2100 Denmark; 4 Department of Cellular and Molecular Medicine, Faculty of Health and Medical Sciences, University of Copenhagen, Blegdamsvej 3, Copenhagen, 2200-DK Denmark; 5 Department of Neurosurgery, Copenhagen University Hospital, Blegdamsvej 9, Copenhagen, DK-2100 Denmark; 6 Department of Medicine, Unit of Microbial Pathogenesis, Karolinska Institutet and Department of Neurosurgery, Karolinska University Hospital, Solna 171 76, Stockholm, Sweden; 7 Department of Pathology, Hospital Na Homolce, Roentgenova 2, 150 30 Praha 5, Czech Republic; 8 Statistics, Bioinformatics and Registry Unit, Danish Cancer Society Research Center, Strandboulevarden 49, Copenhagen, DK-2100 Denmark; 9 Division of Translational Medicine and Chemical Biology, Department of Medical Biochemistry and Biophysics, Science for Life Laboratory, Karolinska Institute, Scheeles vag 2, Stockholm, 17177 Sweden; 10 Department of Radiation Biology, Copenhagen University Hospital, Blegdamsvej 9, Copenhagen, 2100-DK Denmark

Correction to: *Nature Communications*; 10.1038/ncomms13398; published online 15 November 2016

This Article contains an error in the spelling of the author Kjeld Møllgård, which is incorrectly given as Kjeld Møllgaard. The error has not been fixed in the original PDF and HTML versions of the Article.

