# Supplementary material for: BRCA1-regulated RRM2 expression protects glioblastoma cells from endogenous replication stress and promotes tumorigenicity
Source: Nat Commun. 2016 Nov 15;7:13398. doi: 10.1038/ncomms13398 (PMC5116074; doi:10.1038/ncomms13398)
Supplement: Supplementary Information — Supplementary Figures 1-8, Supplementary Tables 1-4 [file ncomms13398-s1.pdf]

Supplementary Figure 1

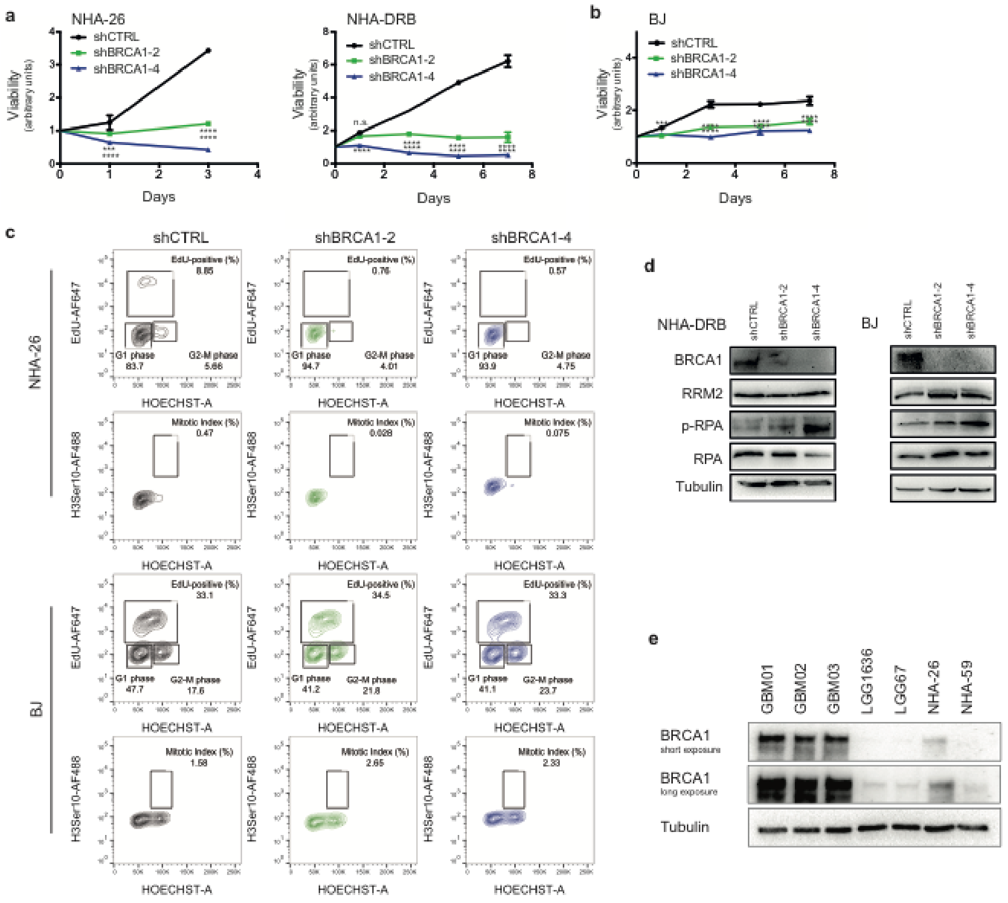

Supplementary Figure 1. Supplementary information to Figure 1.

(**a**) BRCA1 shRNA-mediated knockdown leads to decreased viability of NHA-26 and NHA-DRB cells.

(**b**) BRCA1 shRNA-mediated knockdown leads to decreased viability of BJ cells.

(**c**) FACS analysis of cell cycle profile - the proliferative index (% of Edu-positive cells) and mitotic index (% of $H3^{Ser10}$-positive cells) in NHA-DRB and BJ cells transduced with shCTRL virus or 2 independent BRCA1-targeting shRNAs (shBRCA1-2 and shBRCA1-4).

(**d**) Immunoblot analysis of BRCA1, RRM2, p-RPA and total RPA levels in NHA-DRB and BJ cells transduced with shCTRL virus or 2 independent BRCA1-targeting shRNAs (shBRCA1-2 and shBRCA1-4). Tubulin was used as a loading control.

(**e**) Immunoblot analysis of BRCA1 expression in primary and xenografts cell lines used in our study. BRCA1 SE (BRCA1 short exposure), BRCA1 LE (BRCA1 long exposure). WHO grade IV gliomas (GBM01-03); WHO grade II gliomas (LGG1636 & LGG67); normal human astrocytes (NHA-26; NHA-59). Tubulin was used as a loading control.

Statistical significance was calculated by one-way ANOVA and Tukey's multiple comparisons test in (**a**) and all data are shown as means ±SD and performed as technical triplicates. (*P < 0.05, **P < 0.005, ***P < 0.005, ****P < 0.0001; n.s. represents non-significance)

Supplementary Figure 2

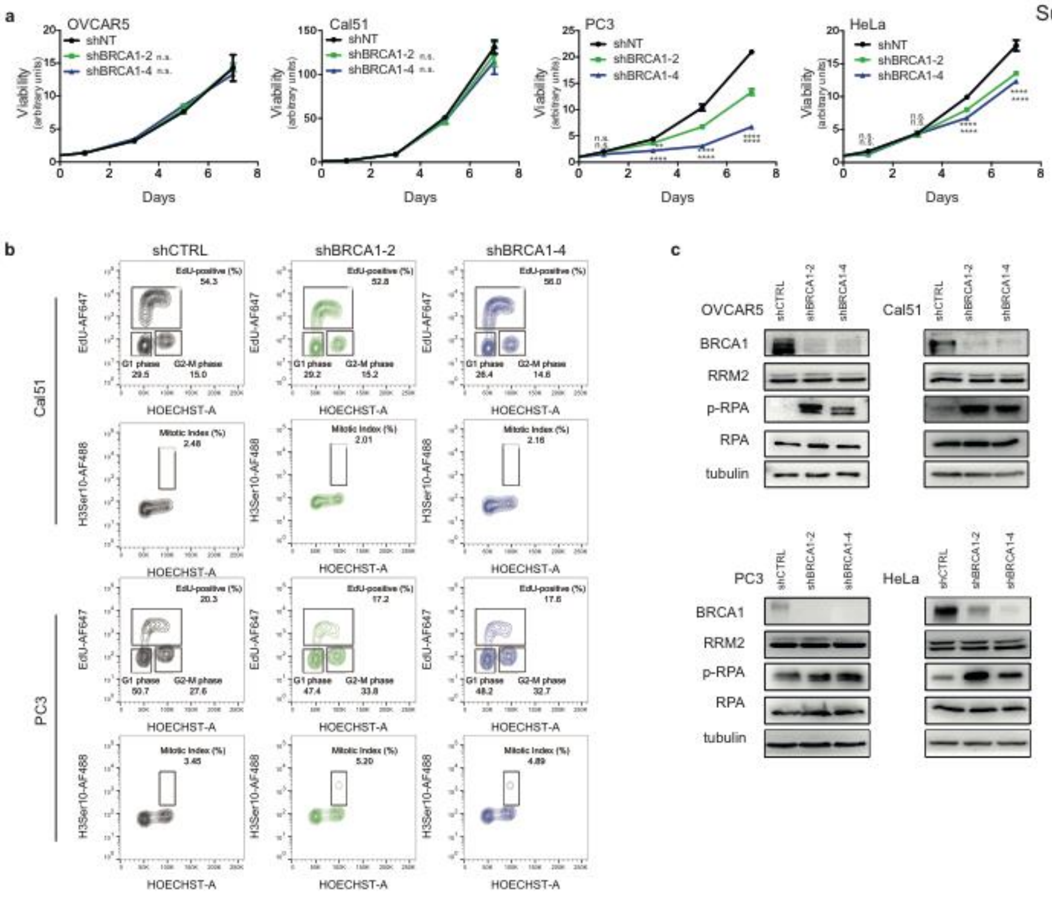

Supplementary Figure 2. BRCA1 loss phenotype in ovarian (OVCAR-5), breast cancer (Cal51), prostate (PC3) and cervical carcinoma (HELA) cells.

(**a**) BRCA1 shRNA-mediated knockdown leads to decreased viability of PC3 and HeLa cells but not OVCAR5 and Cal51 cells.

(**b**) BRCA1 shRNA-mediated knockdown does not effect cell cycle progression of Cal51 cells, but causes reduction in proliferative index (% of EdU$^+$ cells) and G2/M cell cycle arrest in PC3 cells.

(**c**) Immunoblot analysis of BRCA1, RRM2, p-RPA and total RPA levels in OVCAR5, Cal51, PC3 and HeLa cells transduced with shCTRL virus or 2 independent BRCA1-targeting shRNAs (shBRCA1-2 and shBRCA1-4). Tubulin was used as a loading control.

Statistical significance was calculated by one-way ANOVA and Tukey's multiple comparisons test in (**a**) and all data are shown as means ±SD and performed as technical triplicates. (*P < 0.05, **P < 0.005, ***P < 0.005, ****P < 0.0001; n.s. represents non-significance)

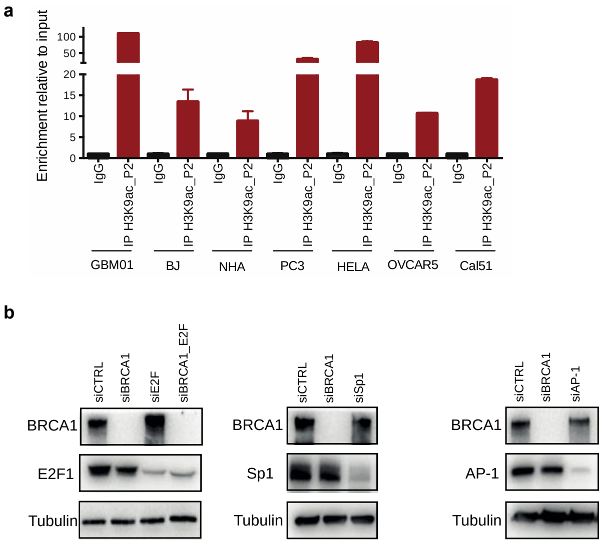

Supplementary Figure 3. Supplementary information to Figure 3h-j and Figure 4b.

(**a**) Chip H3K9ac immunoprecipitation as a positive control for RRM2 promoter Chip analysis in GBM01, BJ, NHA, PC3, HELA, OVCAR and Cal51 cells using primer set P1 and P2.

(**b**) siRNA-mediated knockdown validation for BRCA1, E2F1, Sp1 and AP-1 using immunoblot analysis of BRCA, E2F1, Sp1 and AP-1, respectively. Tubulin was used as a loading control.

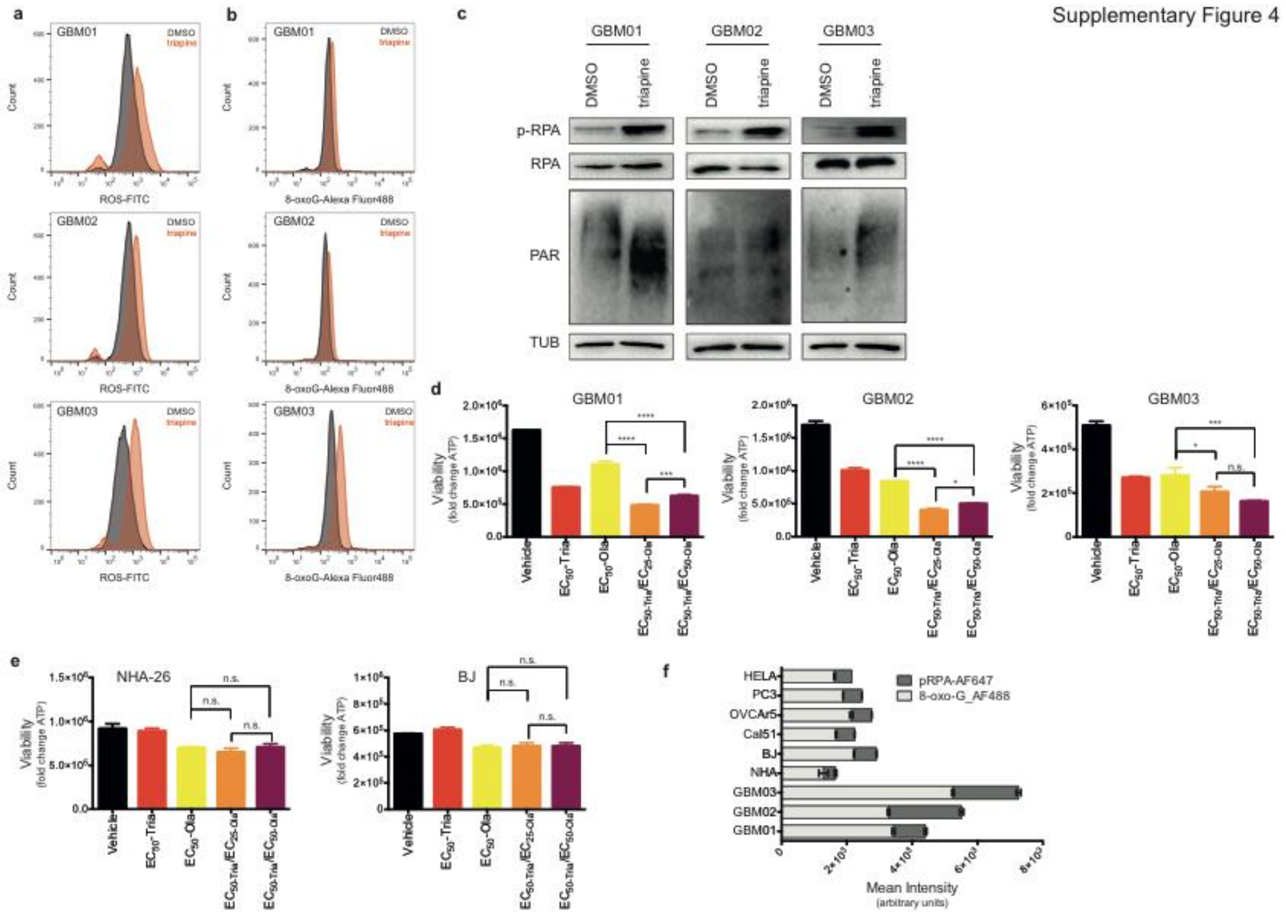

Supplementary Figure 4. Triapine treatment sensitizes GBM cells to olaparib.

(**a**) FACS analysis of ROS induction (ROS-FITC) after EC_{50} triapine treatment of GBM cells compared to DMSO control.

(**b**) FACS analysis of 8-oxo-guanine (8-oxoG-Alexa Fluor488; marker of oxidative DNA damage) positivity after EC_{50} triapine treatment of GBM cells compared to DMSO control.

(**c**) Immunoblot analysis of of p-RPA, RPA and PARylation levels (PAR) in GBM cells treated with DMSO control or triapine (respective EC_{50} for each line).

(**d**) Cell viability of GBM01-03 cells after treatment with respective EC_{50} and/or EC_{25} doses of triapine, olaparib or their combination was assessed by ATP-based assay.

(**e**) Cell viability of NHA-26 and BJ cells after treatment with respective EC_{50} and/or EC_{25} doses of triapine, olaparib or their combination was assessed by ATP-based assay.

(**f**) FACS analysis of mean p-RPA/8-oxo-G_AF488 positivity in cells used in this study.

Statistical significance was calculated by one-way ANOVA and Tukey's multiple comparisons test (**d,e**) and all data are shown as means ±SD and performed as technical triplicates. (*P < 0.05, **P < 0.005, ***P < 0.005, ****P < 0.0001; n.s. represents non-significance)

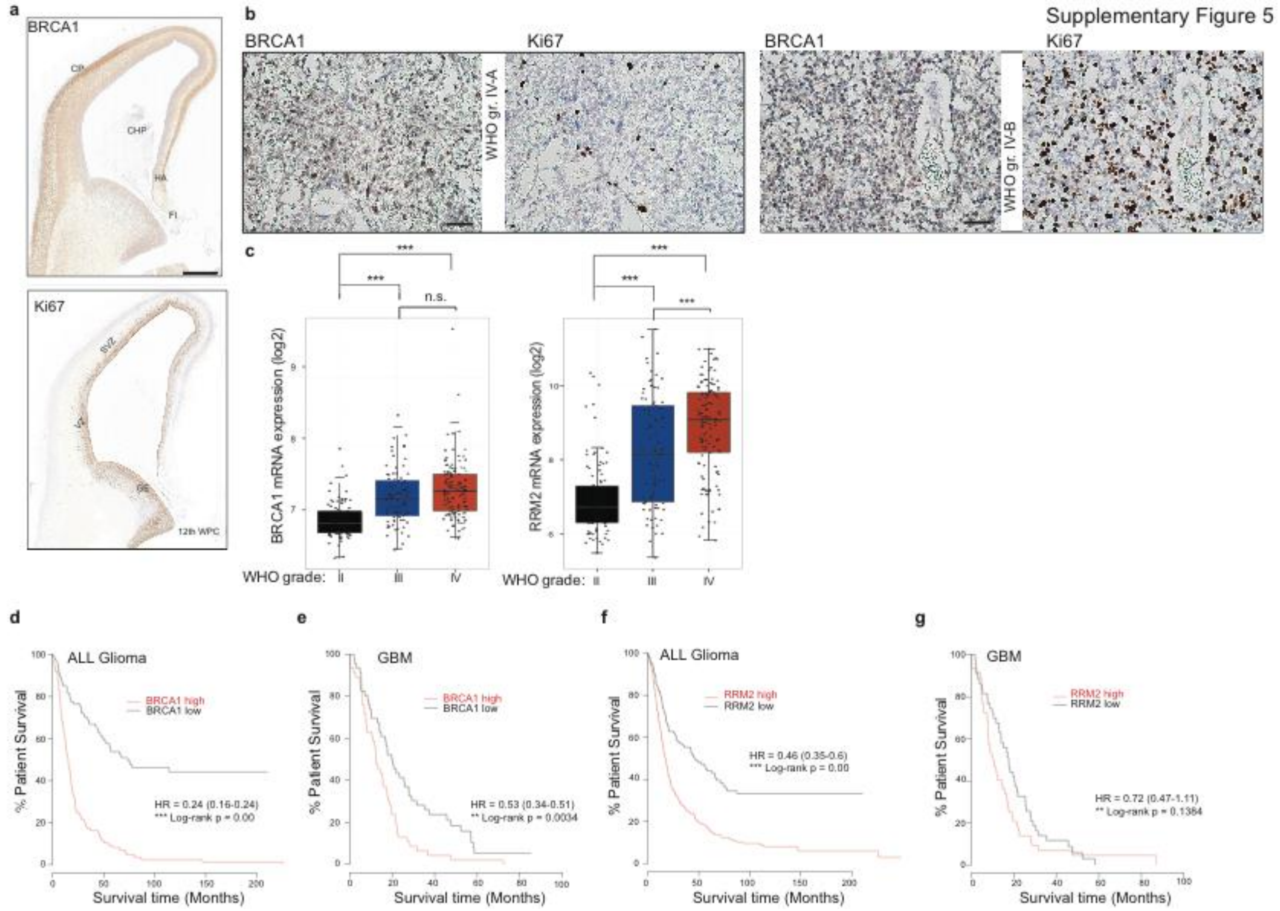

Supplementary Figure 5. Supplementary information to Figure 6.

(**a**) Regional distribution of BRCA1 and Ki67 in horizontal sections of developing forebrain at the level of foramen interventriculare (FI) from a 12th week post conception (WPC) human fetus. Strong BRCA1 immunoreactive labeling depicts the entire cortical plate (CP) and its extension into the amnionic plate in the hippocampal anlage (HA), whereas BRCA1 staining is almost absent in the strongly proliferating ganglionic eminence (GE), ventricular and subventricular zones (VZ and SVZ) (Ki67-immunoreactive labeling). Scale bar 1000 μm.

(**b**) Representative immunohistochemical staining of BRCA1 and Ki67 on parallel sections from 2 representative WHO grade IV gliomas. Scale bar 50 μm.

(**c**) BRCA1 and RRM2 mRNA expression analysis of REMBRANDT data (the National Cancer Institute's repository available via GlioVis) in WHO grade II, III and IV gliomas. (*BRCA1*: WHO grade II versus grade III, $p < 0.0001$; WHO grade II versus grade IV, $p < 0.0001$; WHO grade III versus grade IV AND *RRM2*: WHO grade II versus grade III, $p < 0.0001$; WHO grade II versus grade IV, $p < 0.0001$; WHO grade III versus grade IV, $p < 0.0001$, using Tukey's Honestly Significant Difference test, HSD)

(**d**) Analysis of REMBRANDT data (the National Cancer Institute's repository available via GlioVis) indicates a significant correlation between high BRCA1 expression and poor survival in ALL gliomas (n=47 BRCA1 low; n = 97 BRCA1 high; Log-rank p = 0.00).

(**e**) Same as (**d**) but selected for WHO grade IV patients only (GBM; n = 15 BRCA1 low; n = 60 BRCA1 high; Log-rank p = 0.0034).
(**f**) Analysis of REMBRANDT data (the National Cancer Institute's repository available via GlioVis) indicates a significant correlation between high RRM2 expression and poor survival in ALL gliomas (n=181 RRM2 low; n = 134 RRM2 high; Log-rank p = 0.00).
(**g**) Same as (**f**) but selected for WHO grade IV patients only (GBM; n = 43 RRM2 low; n = 47 RRM2 high; Log-rank p = 0.1384/Wilcoxon p = 0.0405).

Supplementary Figure 6

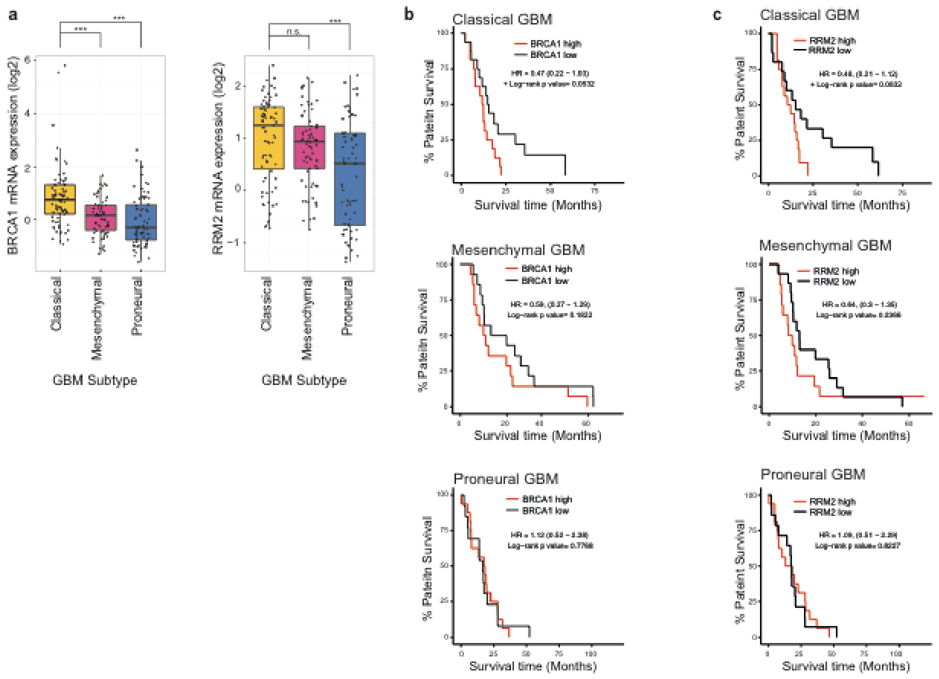

Supplementary Figure 6. Supplementary information to Figure 6.

(**a**) BRCA1 and RRM2 mRNA expression analysis of REMBRANDT data (the National Cancer Institute's repository available via GlioVis) in classical, mesenchymal and proneural subtype of GBM. (***/ $p < 0.0001$ assessed by Tukey’s Honestly Significant Difference test, HSD).

(**b**) Analysis of REMBRANDT data (the National Cancer Institute's repository available via GlioVis) indicates a correlation between high BRCA1 expression and poor survival in classical GBM (n=16 BRCA1 low; n = 16 BRCA1 high; Log-rank p = 0.0532), but no correlation in mesenchymal or proneural GBM.

(**c**) Analysis of REMBRANDT data (the National Cancer Institute's repository available via GlioVis) indicates a significant correlation between high RRM2 expression and poor survival in classical (n=15 RRM2 low; n = 14 RRM2 high; Log-rank p = 0.0832), but no correlation in mesenchymal or proneural GBM subtype.

Supplementary Figure 7

Original immunoblots for indicated figures.

Figure 1

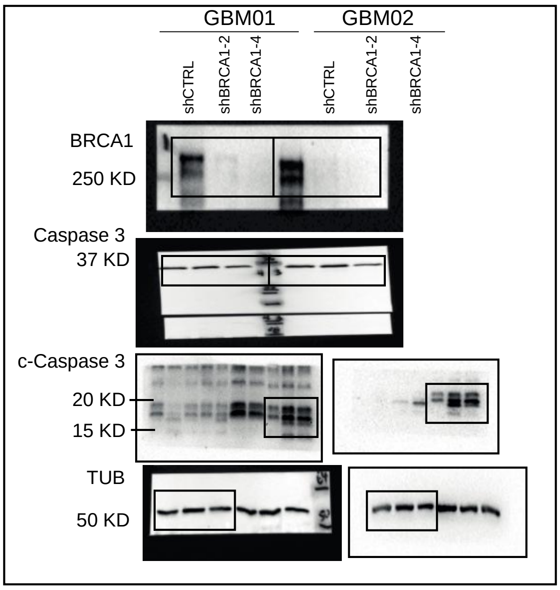

Figure 2

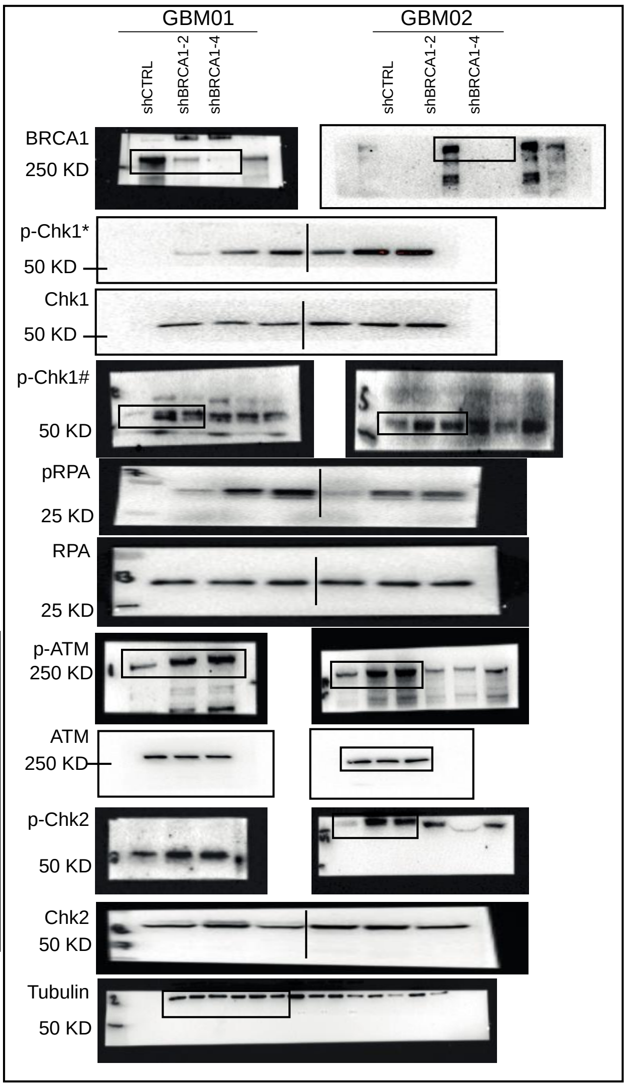

Figure 3

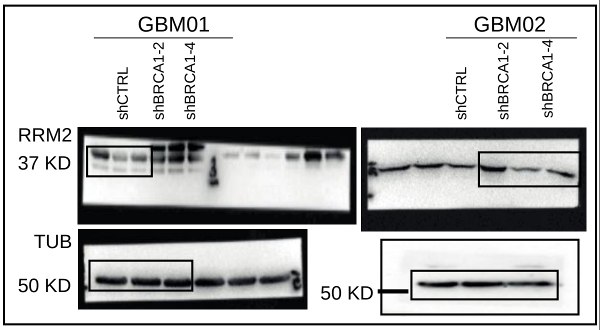

Figure 4

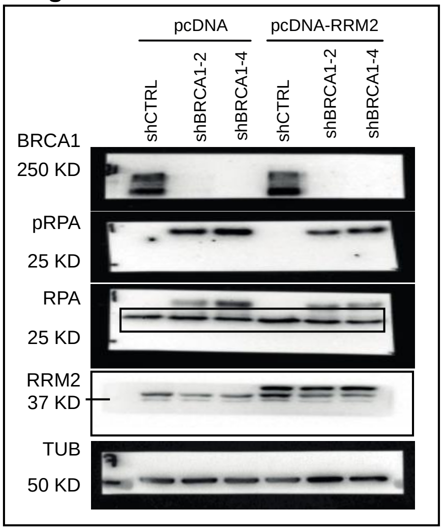

Original immunoblots for indicated figures. Supplementary Figure 8

Supplementary fig 1

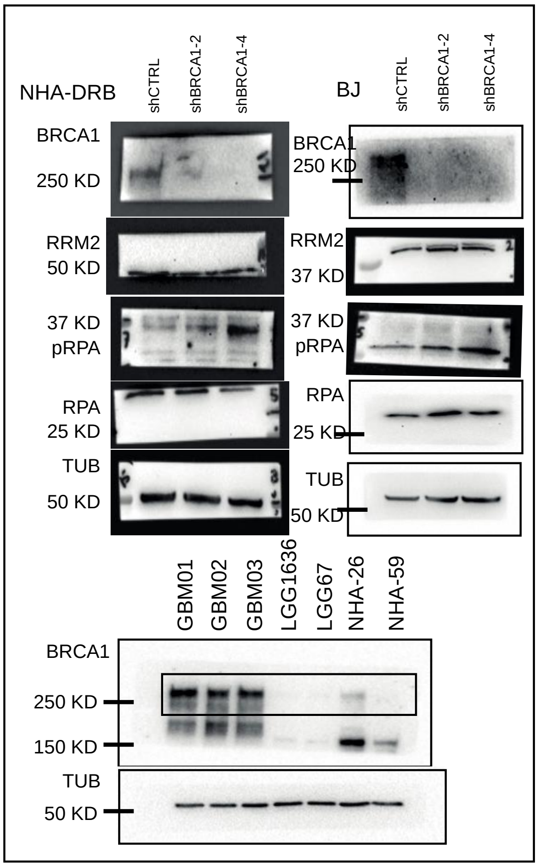

Supplementary fig 2

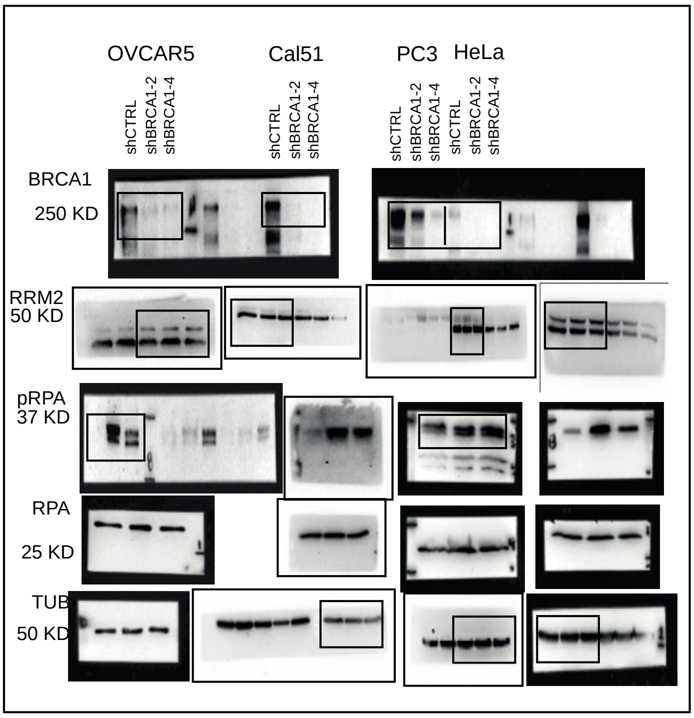

Supplementary fig 3

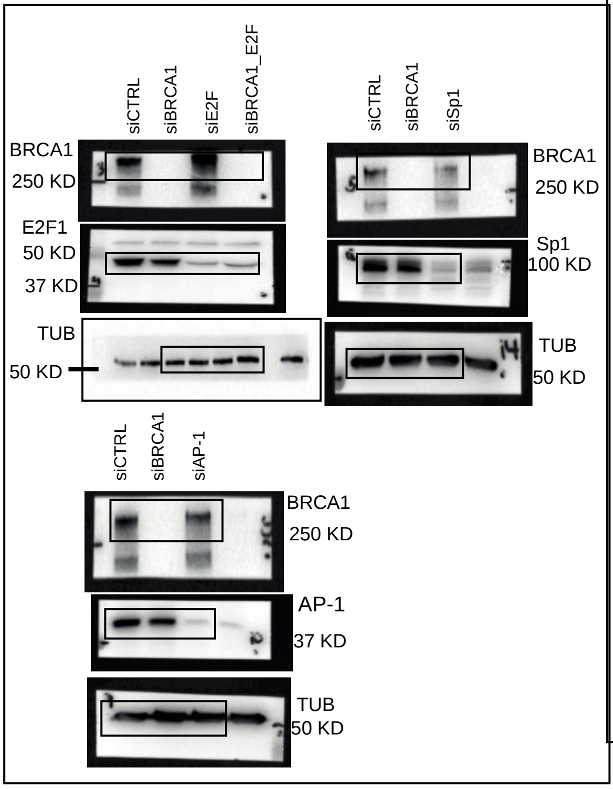

Supplementary fig 4

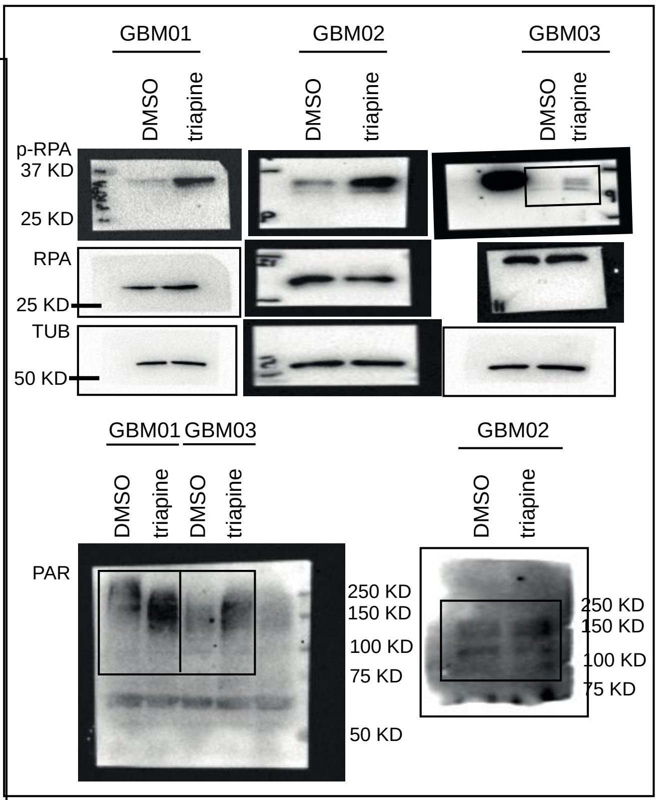

Supplementary Table 1. Statistics and comparison of data in **Figure 3b**. Statistical significance was calculated by Student's *t* test: shCTRL_H2O versus shCTRL_HU (****P < 0.0001); shBRCA1-2_H2O versus shBRCA1-2_HU (****P < 0.0001); shBRCA1-4_H2O versus shBRCA1-4_HU (****P < 0.0001).

GBM01	**Mean fork speed (kb/min)**	**SD**	**SEM**	**n**
shCTRL_H_2O	0,6724	0,3694	0,01652	500
shCTRL_HU	0,3778	0,2074	0,0092	500
shBRCA1-4_H_2O	0,4583	0,2189	0,0098	500
shBRCA1-4_HU	0,2318	0,0944	0,0042	500
shBRCA1-2_H_2O	0,3516	0,1462	0,0065	500
shBRCA1-2_HU	0,1680	0,0694	0,0031	500

Supplementary Table 2. Demographic characteristics of patient cohort

	Number of patients	Male/Female RATIO	Median Age (RANGE)
WHO grade II	50	27/23	39 (21-76)
WHO grade III	20	10/10	51 (28-71)
WHO grade IV	75	45/29	62 (23-62)

Supplementary Table 3. Uni- and multi-variate Cox regression analysis

**Univariate analysis**			
	**HR**	**p - value**	**95% CI**
BRCA1 low	2.52	0.09	0.87 – 7.28
BRCA1 high	7.72	0.00	2.98 – 19.97
RRM2 positive	3.14	0.00	1.82 – 5.42
**Multivariate analysis**			
	**HR**	**p - value**	**95% CI**
BRCA1 low	2.29	0.14	0.75 – 6.98
BRCA1 high	2.34	0.10	0.84 – 6.50
RRM2 positive	1.43	0.23	0.80 – 2.55
WHO gr. III	4.31	0.02	1.22 – 15.23
WHO gr. IV	4.54	0.02	1.29 – 16.02
Age	1.06	0.00	1.04 – 1.08
Sex	0.79	0.34	0.48 – 1.29
Ki67	1.00	0.50	0.99 – 1.02

Supplementary Table 4. List of primary antibodies.

Primary antibodies	Host IgG	Dilution	HIER	Producer	Code number
BRCA1 (IHC)	Rabbit IgG	1:200	Citrate, pH6	Bethyl Laboratories	IHC-00278
BRCA1 (WB)	Rabbit IgG	1:500		Bethyl Laboratories	IHC-00278
Ki-67	Mouse IgG1	1:30	TEG	Dako	M 7240
RRM2 (IHC)	Mouse IgG1	1:400	-	Abcam	Ab57653
RRM2 (WB)	Mouse IgG1	1:500		Abcam	Ab57653
Rad51	Mouse IgG1	1:250		Abcam	ab213
γ-H2AX Ser139	Mouse IgG1	1:1000		Millipore	05-636
p-RPA (Thr21)	Rabbit IgG	1:2000		Abcam	ab61065
RPA	Mouse IgG1	1:1000		Abcam	ab2175
PCNA	Human	1:100		Immuno Concepts	2037
53BP1	Rabbit IgG	1:700		Millipore	MAB3802
Caspase-3	Rabbit IgG	1:1000		Cell Signaling	9662
ATM	Rabbit IgG	1:1000		Cell Signaling	2873
p-ATM (Ser1981)	Rabbit IgG	1:5000		Genetex	GTX61739
Chk1	Mouse IgG1	1:200		Santa Cruz	sc-8408
p-Chk1 (Ser317)	Rabbit IgG	1:300		Cell Signaling	2344
p-Chk1 (Ser345)	Rabbit IgG	1:250		Cell Signaling	2348
Chk2	Mouse IgG1	1:200		DCS, Jiri Bartek	
p-Chk2 (Thr68)	Rabbit IgG	1:200		Cell Signaling	2661
α-tubulin	Mouse IgG1	1:10000		Sigma-Aldrich	T9026-2ML
H3 (Ser10)	Rabbit IgG	1:300		Cell signaling	347580
PAR	Mouse IgG	1:200		GeneTex	3377s
E2F1	Mouse IgG	1:100		DCS, Jiri Bartek	GTX75054
SP-1	Rabbit IgG	1:200		Sigma	HPA001853
AP-1	Rabbit IgG	1:100		Santa Cruz	Sc-045

HIER: Heat Induced Epitope Retrival. Producers: Bethyl Laboratories, INC, Montgomery, USA; Dako, Glostrup, Denmark; Abcam, Cambridge, United Kingdom.
